# The protein subunit of telomerase displays patterns of dynamic evolution and conservation across different metazoan taxa

**DOI:** 10.1186/s12862-017-0949-4

**Published:** 2017-04-26

**Authors:** Alvina G. Lai, Natalia Pouchkina-Stantcheva, Alessia Di Donfrancesco, Gerda Kildisiute, Sounak Sahu, A. Aziz Aboobaker

**Affiliations:** Department of Zoology, University of Oxford, South Parks Road, Oxford, OX1 3PS UK

**Keywords:** Telomerase, TERT, Evolution, Alternative splicing, Planarian, Metazoa

## Abstract

**Background:**

Most animals employ telomerase, which consists of a catalytic subunit known as the telomerase reverse transcriptase (TERT) and an RNA template, to maintain telomere ends. Given the importance of TERT and telomere biology in core metazoan life history traits, like ageing and the control of somatic cell proliferation, we hypothesised that TERT would have patterns of sequence and regulatory evolution reflecting the diverse life histories across the Animal Kingdom.

**Results:**

We performed a complete investigation of the evolutionary history of TERT across animals. We show that although TERT is almost ubiquitous across Metazoa, it has undergone substantial sequence evolution within canonical motifs. Beyond the known canonical motifs, we also identify and compare regions that are highly variable between lineages, but show conservation within phyla. Recent data have highlighted the importance of alternative splice forms of *TERT* in non-canonical functions and although animals may share some conserved introns, we find that the selection of exons for alternative splicing appears to be highly variable, and regulation by alternative splicing appears to be a very dynamic feature of *TERT* evolution. We show that even within a closely related group of triclad flatworms, where alternative splicing of *TERT* was previously correlated with reproductive strategy, we observe highly diverse splicing patterns.

**Conclusions:**

Our work establishes that the evolutionary history and structural evolution of TERT involves previously unappreciated levels of change and the emergence of lineage specific motifs. The sequence conservation we describe within phyla suggests that these new motifs likely serve essential biological functions of TERT, which along with changes in splicing, underpin diverse functions of TERT important for animal life histories.

**Electronic supplementary material:**

The online version of this article (doi:10.1186/s12862-017-0949-4) contains supplementary material, which is available to authorized users.

## Background

In linear eukaryotic chromosomes, telomeres function as protective nucleoprotein structures that guard chromosome ends against the action of nucleases, DNA damage signalling and nucleolytic degradation [1–3]. Telomeric DNA, consisting entirely of guanine-rich tandem repeats, is maintained by the action of the enzyme telomerase. The activity of telomerase was first eluded with the framing of the “end replication problem” by Watson [4] and Olovnikov [5], which noted that the lagging strand nature of DNA replication would lead to progressive shortening of linear eukaryotic chromosomes. In humans, cells in culture have a finite replicative lifespan limiting the number of times a cell can divide, and eventually senesce [6]. This has since been shown to be a key mechanism in protecting against cancer and may also contribute to causing a decline in physiological functions and organismal ageing [7–12]. While together the shortening of chromosome ends and the observation of the “Hayflick limit” led to the notion that telomeres could act as timers of cellular replicative age, it also raised the question as to how chromosomes maintain their ends across generations, through reproduction, development and during the lifespan of long-lived individuals [2, 13–15]. This led to intensive research on both the structure of chromosome ends and a search for the cellular activities that could maintain these structures in light of the “end replication problem”. Eventually, the ribonucleoprotein telomerase was discovered in the ciliated protozoa *Tetrahymena thermophila* [16–20]. Soon after, the catalytic subunit of this enzyme, telomerase reverse transcriptase (TERT) was also discovered and then studied in human cells [21–27].

However, despite telomeres and telomerase providing the solution for the existence of linear chromosomes, it is clear that the huge variation in life histories, particularly amongst metazoans, would have different demands on an end chromosome maintenance system. For example, short-lived and long-lived metazoans would have very different needs for telomerase and telomere maintenance. This is exemplified by comparing ourselves with single celled protists, where human life history in an evolutionary context means that signalling from chromosome ends must achieve a balance between allowing growth and preventing inevitable somatic mutations from causing cancer and death before reproductive age [28, 29]. Subsequent pleiotropic effects of signalling mechanisms associated with telomeres now appear to be central to the human ageing process and age associated pathologies, including processes that are unconnected to telomere length [30–33]. On this basis we would expect to observe telomere biology adaptations to reflect life history specific requirements and therefore variation between different groups. For instance, telomerase levels in the long-lived rodent, the naked-mole rat show stable expression across different life stages [34]. In long-lived birds, telomerase activity is correlated with proliferative potential and the demands of specific organ types, for example birds with long lifespans have high levels of telomerase in their bone marrow [35]. In other species telomerase does not appear to be related to homeostasis and longevity, for example studies in laboratory mice have demonstrated that ageing is unlikely to be caused by telomere shortening alone as aged mice have long telomeres and loss of telomerase has no immediate effect on ageing [11, 36–39]. Unlike mammals that stop growing upon reaching adulthood, certain animals with indeterminate growth such as some fish [40–42], bivalve molluscs [43], sea urchins [44], lobsters [45] and planarians [46] express telomerase through the life cycle.

In addition to its canonical role in telomere maintenance, TERT, the protein subunit of the telomerase enzyme has also been shown to have non-canonical functions [47–50]. Not much is known about whether these functions are conserved or lineage specific. Additionally, *TERT* transcripts in many animal species, including vertebrates, insects, nematodes and planarians are alternatively spliced [46, 51–55] and spliced variants have also been shown to have non-canonical roles, for example in cell proliferation [56, 57]. *TERT* splicing dysregulation is present in many cancers and it is thought that human *TERT (hTERT)* splicing could be manipulated for therapeutic purposes [58, 59]. While only a little is known about *TERT* alternatively spliced (AS) variants in animals, this represents a potential evolutionary mode for lineage specific life history adaptation, and thus broader assessment of *TERT* alternate splicing is required particularly in lineages spanning short evolutionary time frames.

Given the importance of telomerase function in lineage-specific life history traits, it is surprising that an in depth understanding of the evolutionary history of TERT in the Metazoa is currently lacking from the literature. Here, we make a detailed study of TERT across the Animal Kingdom using extant data to look for evidence (or lack) of patterns of TERT protein and alternative splicing evolution. We identify TERTs across animal phylogeny and perform within and between phyla comparison of the structure of this gene. We are able to confirm the domain content of the ancestral TERT at the base of the Animal Kingdom and then we are able to show dynamic evolution of the protein in different lineages. This includes both the loss of canonical motifs and the invention of lineage specific domains, for example the invention of novel C-terminal extension and N-terminal linker motifs specific to the vertebrate lineage. In some cases we hypothesized that novel motifs may simply compensate for lost canonical motifs, and in others they may relate to lineage specific activities and interactions that remain to be discovered. Next we use available data to assess the levels of alternative splicing, implicated in non-canonical TERT function in humans and described these in different taxa. We find evidence that alternative splicing is likely to be common feature of some lineages, but is not broadly conserved with respect to splicing patterns. Finally, we study the evolution of splicing in one particular related group of animals, the free-living Tricladida planarians [60–62]. We find that it evolves relatively rapidly in this group of highly regenerative animals, suggesting that study of alternative splicing over shorter evolutionary timescales may be required to understand adaptive non-canonical roles of TERT. Taken together our work highlights a previously unappreciated evolutionary and likely functional diversity in this core eukaryotic protein. We conclude that telomere biology, a core synapomorphy of eukaryotes, has undergone significant changes in different lineages and some of this is through the evolution of the TERT protein subunit of telomerase itself.

## Results and discussion

### TERT distribution across the animal kingdom and reconstruction of the ancestral TERT domain structure

**Fig. 1:**
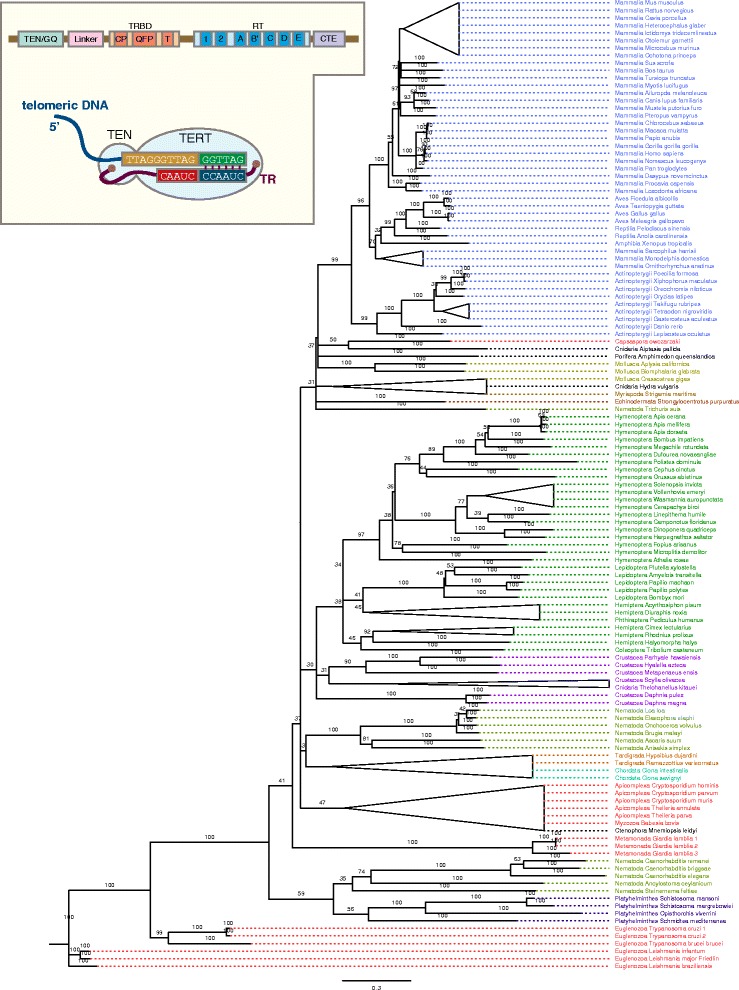
Evolution of TERT across the Animal kingdom. Phylogenetic tree of TERT sequences from representative metazoan phyla, early-branching metazoans and unicellular relatives of metazoans constructed using the maximum-likelihood method. Bootstrap support percentages from 1000 replicates are labelled at the nodes. *Scale bar* denotes substitutions per site. Branches that contained less than 30% boostrap replicates were collapsed. Full list of species used and accession numbers can be obtained from Additional file 1: Table S2. Figure inset shows a schematic diagram of the canonical telomerase enzyme bound to the telomeric DNA substrate and the telomerase RNA template. The domain structure of TERT is also shown: the TEN domain with the GQ motif, the TRBD domain with the CP, QFP and T motifs, the RT domain with the 1, 2, A, B′, C, D and E motifs, the N-terminal linker and the CTE region

While TERT proteins perform core functions across eukaryotes, not much is known about how conserved or not TERT protein structure is between different metazoan lineages. In order to address this, we set out to define the structure of TERT in the metazoan ancestor and use this as a basis for comparison amongst animals. We searched for TERT orthologs from publicly available genomic and transcriptomic datasets and retrieved 135 *TERT* sequences representative of different metazoans and unicellular eukaryotes. We constructed a maximum-likelihood tree with the TERT sequence from Euglenozoa as outgroup. In many cases but not all, we observed that tree topology reflected the appropriate phylogenetic relationships between phyla (Fig. 1).

#### TERT is found in unicellular relatives of metazoans and early-branching metazoans

Metazoans are related to unicellular lineages from the holozoan clade: filastereans, ichthyosporeans and choanoflagellates [63–67]. While we could identify *TERT* from *Capsaspora owczarzaki*, we could not find evidence for *TERT* orthologs in either the choanoflagellates *Monosiga brevicollis* or *Salpingoeca rosetta*. Despite this, both *M. brevicollis* [68] and *S. rosetta* [69] have the 5′-TTAGGG(n)-3′ telomeric repeat. Based on this finding, a canonical TERT is predicted to be present [68]. The observed absence could reflect incomplete genome sequences or, more likely given the absence from two different choanoflagellate genomes, that TERT primary sequence has evolved beyond detection or a related reverse transcriptase activity has instead taken up the role of TERT. From the genome sequences of other unicellular parasitic protozoans, putative *TERT* sequences have been predicted [70–77] and our phylogenetic analyses confirms that these predictions are all canonical *TERT* proteins, so it seems likely that loss of a recognisable TERT in choanoflagellates is a derived character rather than the alternative hypothesis of independent evolution of TERT like proteins in different eukaryotic lineages (Fig. 1). *TERT* sequences from early-branching metazoans reported to date were from the genomes of *Hydra magnipapillata* and *Nematostella vectensis* [78, 79]. The genomes of the sponge *Amphimedon queenslandica* [80]*,* comb jelly *Mnemiopsis leidyi* [81]*,* myxosporean *Thelohanellus kitauei* [82] and sea anemone *Aiptasia pallida* [83] have been sequenced and from these genomes we identified new *TERT* sequences for comparative analysis (Fig. 1; Additional file 1: Table S2). While TERT proteins have been identified in many bilaterian phyla [21–25, 34, 46, 52, 55, 84–94], we additionally identified novel TERT sequences that have not been reported elsewhere. These include TERT orthologs from nematodes species of the classes Secernentea, Chromadorea, Enoplea and Rhabditea and multiple orthologs from Insecta, Actinopterygii, and Mammalia (Additional file 1: Table S2). We also identified novel *TERT* sequences from parasitic platyhelminthes from the Trematoda class, from the newly sequenced tardigrade genomes of *Hypsibius dujardini* [95] and *Ramazzottius varieornatus* [96] and the crustacean *Parhyale hawaiensis* [97] (Additional file 1: Table S2).

#### TERT is absent from the bdelloid rotifer

Despite the ubiquity of TERT amongst metazoans, its presence is not universal. TERT has been reported to be absent from some animal species; one prominent example being the loss of TERT in Diptera before divergence from Siphonaptera [98]. Like in dipterans, we failed to identify *TERT* in the bdelloid rotifer *Adineta vaga*, suggesting the loss of TERT from this group of long-term asexual species [99, 100]. In the absence of TERT and canonical telomere repeats, *Drosophila melanogaster* uses a retrotransposon-based telomere maintenance in which telomere-specific retrotransposons are reverse transcribed onto chromosome ends [101]. Three non-Long Terminal Repeat (LTR) retrotransposons, *HeT-A* [102, 103]*, TART* [104, 105] and *TAHRE* [106] are specifically used for telomere maintenance in this species. The bdelloid chromosome ends also have specialised telomere-associated retrotransposons, such as the *Hebe* transposon [107] and sub-telomeric reverse transcriptase (RT)-related genes [108]. *Penelope*-like elements, which lack the endonuclease domain and are located in sub-telomeric regions and are found in two bdelloid rotifer species *A. vaga* and *Philodina roseola* [109]. Although no evidence yet directly links non-LTR transposons to end chromosome maintenance in *A. vaga,* it seems plausible that in the absence of *TERT*, bdelloid rotifers may employ retrotransposon-based mechanisms for telomere maintenance.

#### The ancestral structure of TERT in metazoans is likely to possess a complete complement of canonical motifs

**Fig. 2:**
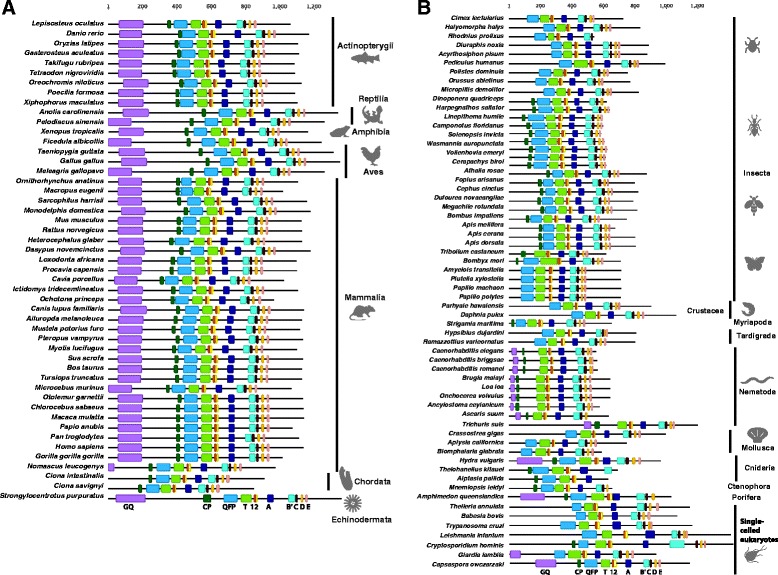
Taxa-specific modifications of TERT domains. TERT canonical motifs from representative metazoan phyla, early-branching metazoans and unicellular relatives of metazoans were annotated in colour codes: TEN domain GQ motif (*purple*); TRBD domain CP (*dark green*), QFP (*blue*) and T (*light green*) motifs; reverse transcriptase domain motifs 1 (*brown*), 2 (*yellow*), A (*dark blue*), B′ (*sky blue*), C (*black*), D (*orange*) and E (*pink*). **a** Deuterostome TERT canonical motifs. **b** Protostome, early-branching metazoans and unicellular eukaryotes canonical motifs. Motif annotations were drawn according to scale based on multiple sequence alignments performed using MAFFT

The TERT protein contains three canonical domains and 11 motifs: 1) a telomerase essential N-terminal (TEN) domain with the GQ motif, 2) a telomerase RNA-binding (TRBD) domain with the CP, QFP and T motifs and 3) a reverse transcriptase (RT) domain with the 1, 2, A, B′, C, D and E motifs (Fig. 1) [9, 110]. Beyond these canonical domains and motifs, there is a flexible linker region called the N-terminal linker between the GQ and CP motifs and a C-terminal extension region that starts after the last E motif (Fig. 1) [9]. The T motif allows for correct distinction of TERT family proteins from other prototypical RT proteins and we find that this motif is present in all species used in this study (Fig. 2). To confirm the ancestral metazoan structure of TERT, we performed a cross-species comparison of all TERT proteins (Fig. 2). We inferred that TERT in the metazoan ancestor possesses all 11 canonical motifs outlined above based on the observation that a complete complement of canonical motifs is present in the unicellular filasterean *C. owczarzaki* and the cnidarian *Hydra vulgaris* (Fig. 2). Within Bilateria taxa considered here, only vertebrates and the echinoderm *Strongylocentrotus purpuratus* have retained all 11 motifs (Fig. 2), while all protostomes have lost one or more canonical motifs suggesting broad differences in how TERT executes its core function may exist in metazoans.

### Taxa-specific divergence of canonical TERT motifs

We next examined evolution of TERT canonical motifs in detail across taxa. Sequence analyses of 11 TERT canonical motifs revealed that the TEN (GQ motif) and TRBD (CP, QFP and T motifs) domains have high sequence conservation across their whole lengths (Additional file 2: Figure S1).

### TERT sequence divergence in unicellular relatives of metazoans

Amongst unicellular eukaryotes considered here, only *C. owczarzaki* TERT has all 11 motifs (Fig. 2). An independent study reported that TERT in *Leishmania amazonensis* also contained all 11 motifs [76]. However, from our sequence alignment analyses, we were unable to identify clearly defined GQ motifs in *Leishmania sp., Trypanosoma sp., Theileria sp., Babesia sp.* and *Cryptosporidium sp.* (Additional file 3: Figure S2). The observation of GQ in *L. amazonensis* might be an alignment artefact because from their alignment, only eight out of 244 amino acid residues are found to be in common with other TERTs [76]. We also failed to find the CP motif in this group of unicellular eukaryotes, except for *C. owczarzaki* and *Cryptosporidium sp.* (Additional file 3: Figure S2). It was reported *Giardia lamblia* lacks the T motif [111]. However, this could be due to the use of evolutionarily distant TERT sequences from plants, *Caenorhabditis elegans* and yeast, for the alignments in their earlier study. We retrieved three TERT sequences for the parasitic protozoan *G. lamblia*, obtained from different animal isolates [112]. Sequence alignment revealed that they share only 72% identity. From an alignment of these *G. lamblia* TERTs with other protozoans, we were able to identify a putative T motif, suggesting that it hasn’t been lost. The *G. lamblia* T motif, however, has eight extra residues not present in other unicellular eukaryotes (Additional file 3: Figure S2). Apart from *C. owczarzaki* and *G. lamblia,* it appears that the TEN domain has diverged substantially in unicellular eukaryotes with poor conservation to the canonical GQ motif. Within kinetoplastid protozoans from the genera *Leishmania* and *Trypanosoma*, we identified a conserved block of residues, (I/V)QQRVxLQF, between the QFP and T motif. Considering that the divergence time of *Trypanosoma sp.* from other trypanosomatids including *Leishmania sp.* is well over 300 million years ago (m.y.a) [113], it is likely this block of residues in TERT is an example of a lineage specific motif.

#### TERT sequence divergence in early branching metazoans

Amongst early branching metazoans, TERT sequences from the comb jelly *M. leidyi* and two cnidarians, *A. pallida* and the myxozoan *T. kitauei* appear to be incomplete because their sequences do not extend beyond reverse transcriptase canonical motifs D, B and A respectively (Fig. 2). TERT from the sponge *A. queenslandica* appears to be complete but entirely lacks the E motif. The GQ motif is collectively absent from the comb jelly and cnidarians *T. kitauei* and *A. pallida*, but not from *H. vulgaris,* indicating that multiple independent losses of GQ occurred amongst early branching metazoans (Fig. 2).

#### TERT sequence divergence in bilaterians

Within Deuterostomia, vertebrates and *Strongylocentrotus purpuratus* have retained all 11 conserved motifs while *Ciona sp.* lack the GQ motif (Fig. 2) [90]. The canonical GQ motif has also been lost from protostomes, except from the nematode lineage (Fig. 2, Additional file 4: Figure S3). In addition to the free-living *Caenorhabditis sp.* [52], we find that other nematodes have also retained the GQ motif (Additional file 4: Figure S3). As previously reported we find that the early branching nematode *Trichinella spiralis* TERT has all 11 canonical TERT motifs [91], so the ancestor of nematode likely possessed all canonical motifs. Studies on human cell lines have shown that within the GQ motif, an additional region known as ‘dissociates activities of telomerase’ (DAT), is essential for TERT activity [114]. Although nematodes have retained the GQ motif, the DAT region within this motif appears to have lost multiple important residues (Additional file 4: Figure S3). The DAT region was proposed to be involved in other aspects of in vivo telomere elongation because mutations in this region have no direct effect on human TERT multimerization, nuclear targeting or template binding [114]. It appears that either nematodes do not have sensu stricto DAT regions, or that the GQ motif in protostomes, including nematodes, have diverged significantly that sequence conservation with the deuterostome GQ can no longer be detected (Additional file 4: Figure S3). The GQ motif in yeast, human cell lines and *T. thermophila* plays a role in repeat addition processivity [9, 114–117]. It remains to be determined, however, whether the loss or sequence divergence of GQ in protostomes bears any functional implications in species-specific life histories. From the crystal structure of the red flour beetle *Tribolium castaneum* TERT, it was shown that although GQ is absent in this species, remaining motifs (CP and T) in the N-terminal region of TERT not only share high degrees of structural conservation with *T. thermophila* TERT [118, 119] but also conferred in vitro activity [120]. Therefore, even in the absence of a canonical GQ sequence across protostomes (Fig. 2), structural and functional conservation of TERT N-terminal region may persist in these phylogenetic groups. The second TERT canonical motif CP has also been also lost in some protostomes: e.g. in molluscs and tardigrades (Fig. 2). Similarly, most arthropods lack the CP motif, but several exceptions exist: the centipede *Strigamia maritima*, hymenopterans (ants, sawflies, bees but not wasps) and *T. castaneum* [118] have retained the CP motif (Fig. 2; Additional file 5: Figure S4). Other TERT motifs are maintained in metazoan taxa.

We next performed functional divergence analyses on monophyletic clusters from the TERT phylogeny using the DIVERGE programme [121]. A coefficient of functional divergence (θ) value that is significantly different from zero, as indicated by the z-score (MFE z-score) for the model-free method (MFE θ) or the likelihood ratio test (LRT θ) for the maximum likelihood estimate (θ ML), suggests evidence for functional divergence between two clusters. MFE θ and θ ML estimates appeared to be statistically significant for five pairwise comparisons: Euglenozoa/Nematoda (MFE θ = 0.56, *P* value <0.01, θ ML = 0.55, LRT θ = 16.54), Actinopterygii/Euglenozoa (MFE θ = 0.79, *P* value <0.01, θ ML = 0.74, LRT θ = 22.63), Mammalia/Crustacea (MFE θ = 0.54, *P* value <0.01, θ ML = 0.66, LRT θ = 33.55), Mammalia/Euglenozoa (MFE θ = 0.76, *P* value <0.01, θ ML = 0.76, LRT θ = 22.28) and Mammalia/Apicomplexa (MFE θ = 0.61, *P* value <0.01, θ ML = 0.69, LRT θ = 35.25; Additional file 6: Table S1). Future experimental validations will be required to investigate these results in detail.

### Identification of novel lineage-specific motifs in TERT

TERT proteins from most organisms possess two highly variable regions that are not part of the 11 canonical motifs: the C-terminal extension (CTE) and the N-terminal linker region [9]. The CTE region starts after the last canonical E motif, and despite poor sequence homology of CTEs between human, *T. castaneum* and *T. thermophila*, it is thought to constitute the RT ‘thumb’ that is made up of three α-helices crucial in template binding and TERT processivity [118, 122–124]. The mechanistic significance of the CTE has remained elusive until recently, when the CTE was shown to be directly involved in differential binding of DNA and a mutated version of the CTE resulted in defective DNA binding and faster DNA dissociation rates [125].

#### CTE regions in unicellular relatives of metazoans and early branching metazoans have regions with high conservation

Since the CTE appears to play an important role despite having poor sequence conservation we examined whether this region has evolved to harbour lineage-specific amino acid conservation. We extracted regions after the last canonical E motif from 96 species representing various taxa and investigated these as potential CTEs. Not all species have CTE regions because TERT proteins in some animals do not extend beyond the E motif. We show that unicellular eukaryotes and two early branching metazoan species, *H. vulgaris* and *A. queenslandica* possess CTE regions (Additional file 7: Figure S6A). Alignment of unicellular eukaryotes CTE regions revealed that blocks of conservation exist within different protozoan lineages, although not between unicellular species and human TERT (Additional file 7: Figure S6C). *Crytosporidium sp.* CTE regions are considerably shorter than those of other protozoans. Kinetoplastid protozoans, *Leishmania sp.* and *Trypanosoma sp*., have a block of conserved residues made up of 48 amino acids at the end of their TERT proteins (Additional file 7: Figure S6C). In other early-branching metazoans, although the parasitic cnidarian *T. kitauei* and the comb jelly *M. leidyi* lack CTE regions, we identified some conserved residues in CTE regions when aligning these regions from *A. queenslandica* and *H.vulgaris* (Additional file 7: Figure S6A).

#### Taxa specific conservation of CTE regions exist in bilaterians

Within bilaterians, it has been reported that nematodes from the *Caenorhabditis* genus have some of the shortest TERT proteins and appear to have lost their CTE structure [52, 111]. We observe that this is also true for the parasitic roundworm *Ancylostoma ceylanicum* where the C-terminal region beyond the E motif only has 42 amino acid residues (Additional file 7: Figure S6E). We discovered that most parasitic nematodes from the Chromadorea and Secernentea classes possess intact CTE regions (Additional file 7: Figure S6E). In addition, we show that filarial nematodes *B. malayi*, *O. volvulus* and *L. loa* share a RIAVL(R/K)FLKASLLEKYR(M/V motif (Additional file 7: Figure S6E). The observation that a CTE is present in most parasitic nematodes but not the free-living *Caenorhabditis sp.* may reflect adaptation of TERT to their very different life histories; i.e. some parasitic nematodes have long generation times and life expectancies and are able to survive for years in their hosts [126]. CTE structures in Arthropoda varied markedly and we performed separate alignments for insects and crustaceans (Additional file 7: Figure S6D and F). We identified two crustacean specific motifs within the CTE region RL(K/Q)x(I/V) and R(L/F)xAL (Additional file 7: Figure S6F). Within insects, ants lack the CTE completely as TERT proteins within this lineage do not extend beyond the E motif (Additional file 7: Figure S6D). Lepidopterans have additional residues at the extreme ends of their TERT proteins conserved within this lineage only; a lepidopteran-specific CTE contains 32 to 36 residues (Additional file 7: Figure S6D). Within Deuterostomia, we annotated highly conserved CTE motifs in tetrapods and fishes (Additional file 7: Figure S6G).

Our findings suggest that the ancestor of metazoans possessed a CTE and that while this feature has been largely retained in most animal lineages, it has been lost multiple times. We have demonstrated that these CTE regions can be incredibly diverse across animal phyla, and we show that novel motifs exist in different lineages. It seems likely that these lineage specific motifs in the CTE region would have significant roles, since functional studies in *T. thermophila*, yeast and humans have revealed the importance of CTE for TERT catalytic activity related to enzyme multimerization, processivity and DNA binding [124, 125, 127–129].

#### Conserved residues within the N-terminal linkers of unicellular relatives of metazoans and early-branching metazoans

We next investigated the N-terminal hypermutable linker (hereafter referred to as the linker) of TERT that is defined as the region between the GQ motif and CP motif. The linker region, although evolutionarily divergent, has been shown to be biologically essential for the function of TERT. In vertebrates, the association between TERT and telomerase RNA (TR) is mediated by the VSR motif in the linker region that binds the activation domain of TR [130, 131]. The puffer fish *Takifugu rubripes* has an additional short alpha-helix upstream of the VSR motif, known as the TFLY motif that is also implicated in binding the template boundary element [119, 132], which is a precise region within the RNA template that is defined by structural elements identified in *T. thermophila*, *Kluyveromyces lactis* and mammals [133–136]. Amongst early-branching metazoans, we found pockets of conservation in the linker region; the motif LxxAIF is present in three out of five early-branching metazoan species (Additional file 8: Figure S7A). Unicellular eukaryotes have very divergent N-terminal linkers between phyla but some conservation within each protozoan lineage, the kinetoplastids (*Leishmania sp.* and *Trypanosoma sp.*) have the ALxR(T/L)(D/N)V(P/S)RxxL motif and the piroplasmids (*Babesia sp.* and *Theileria sp.*) have the LLxGLF motif (Additional file 8: Figure S7C).

#### Vertebrates have a distinct VL region within their N-terminal linkers

We performed an alignment of the linker regions from representative deuterostome phyla and observed that linker regions have poor conservation and they do not align well. Separately, an alignment of linkers from vertebrates alone revealed that vertebrates share a well-conserved block of 91 amino acid residues directly upstream of the CP motif, which we named the Vertebrata-specific N-terminal linker (VL; Additional file 8: Figure S7F). Within this VL region we noticed that class specific motifs exist. According to the molecular timescale for vertebrate evolution, mammals diverged from birds 310 m.y.a. and marsupials from placental mammals at 173 m.y.a. [137]. Teleosts and tetrapods diverged approximately 450 m.y.a. after the vertebrate genome duplication [138]. Such long evolutionary distances enabled us to distinguish conserved motifs from neutrally evolving sequences. For example, we demonstrated that ray-finned fishes have the FIRTLGFLY and RRxQGxD motif, birds have the NQSL motif and marsupials have the HLFxxKGDPxQQ motif (Additional file 8: Figure S7F). It seems highly likely that these conserved motifs have functional roles that will be important for TERT function in each of these lineages.

#### Protostomes have truncated N-terminal regions

Extending our analyses to protostomes, we observed that protostomes generally have truncated N-terminal regions. Because arthropods and lophotrochozoans lack the GQ motif altogether, we extracted sequences upstream of the CP motif (for species that have CP motifs) or the QFP motif (for species without the CP motif) as putative linker regions (Additional file 8: Figure S7B, D and E). Insects, including ants, bees and lepidopterans exhibit long stretches of conserved residues (Additional file 8: Figure S7D). Crustaceans from the classes Malacostraca and Branchiopoda share both FPxxHILS and ILxxNxG motifs in their N-terminal linker regions (Additional file 8: Figure S7A). Amongst nematodes, most species with *Trichuris suris* as the exception (Fig. 2), have truncated N-termini upstream of the CP motif. Nevertheless, some conservation exists within this region, for instance nematodes share a IGxxNxxxx(L/V) motif (Additional file 5: Figure S4A). Overall, although the functional significance of the N-terminal linker is unclear beyond vertebrates, it is apparent that the linker sequence can provide additional surface areas to allow conformational flexibility between the N-terminus and the rest of the TERT protein [9]. It is likely that the conserved linker modifications within different lineages confer some but yet unknown essential biological functions.

### TERT exhibits conservation of intron-exon structure and pan-metazoan regulation by alternative splicing

#### Intron-exon structure of TERT in metazoans

**Fig. 3:**
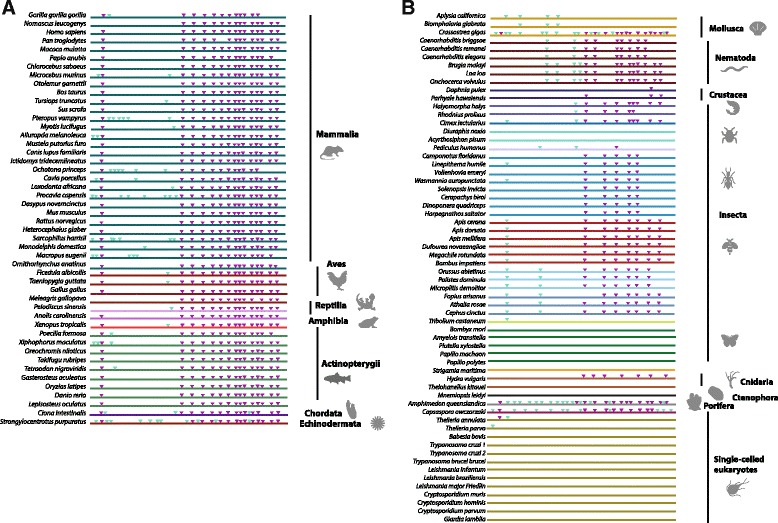
*TERT* exon-intron structure is conserved across the Animal kingdom. Schematic diagram depicting the intron positions mapped on the TERT orthologs relative to intron positions in human TERT (hTERT). **a** Deuterostome *TERT* intron positions. **b** Protostome, early-branching metazoans and unicellular eukaryotes intron positions. *Purple triangles* represent conserved introns (relative to hTERT intron positions) and *green triangles* represent species-specific introns

*TERT* genes are the only example of reverse transcriptase-related genes that have defined biological functions and the preservation of exon-intron structure over long evolutionary periods [24, 108]. We wished to assess changes in both intron structure and alternative splicing of *TERT* across animals. We performed cross-phyla comparison of intron positions within the *TERT* gene family. We defined intron positions according to methods described by another study that compared introns across large evolutionary distances [139]. Intron positions were annotated on protein sequences, where data were available, by determining the boundaries of each coding exon. TERT protein sequences with annotated intron positions were aligned and conserved intron positions were defined as shared intron placements at equivalent positions with hTERT that has 15 introns. With the exceptions of *C. owczarzaki* and *Theileria sp.*, *TERT* in unicellular eukaryotes were found to lack introns altogether (Fig. 3). At the base of Eumetazoa, *TERT* from the comb jelly *M. leidyi* and cnidarian *T. kitauei* are also intronless while *H. vulgaris* and *A. queenslandica* have seven and 29 introns respectively. All seven introns in *H. vulgaris* have shared positions with *hTERT* while 14 out of 29 intron positions in *A. queenslandica* are shared with *hTERT*. These data suggest great flexibility in *TERT* intron content early in metazoan evolution (Fig. 3). Within deuterostome taxa, we observed that introns in 41 out of 48 species share equivalent positions with all 15 *hTERT* introns (Fig. 3a). Many deuterostome *TERTs* have additional introns in positions not conserved with *hTERT*. For example, marsupials have between one to six additional introns in non-conserved positions and the sea urchin *Strongylocentrotus purpuratus* has 11 additional non-conserved introns (Fig. 3a). We observed that *TERTs* in protostomes generally have lower intron numbers (Fig. 3b). Nematodes have nine to 14 introns and they share seven to 10 intron positions with *hTERT* (Fig. 3b). *TERT* in crustaceans are surprisingly intron poor, despite reports that the genome of *Daphnia pulex* has one of the highest intron densities [140]. *D. pulex* and *P. hawaiensis* have only one and three introns respectively, and all introns have shared positions with *hTERT* (Fig. 3b). *TERT* genes in the Hemiptera suborder Sternorrhyncha have undergone unique genomic changes compared to other hemipteran insects; *TERT* in aphids and lepidopterans have lost all introns (Fig. 3b). Within lophotrochozoa, only the pacific oyster is intron rich; the oyster has 27 introns and 14 of these share equivalent positions with *hTERT* (Fig. 3b). Other molluscs are intron poor; *Aplysia californica* and *Biomphalaria glabrata* have five and three introns respectively, none of which have positions shared with *hTERT* (Fig. 3b).

#### Evidence of alternative splicing of TERT in bilaterians

Introns provide the opportunity for creating new gene products via alternative splicing mechanisms. *TERT* alternatively spliced products have been implicated in non-canonical functions in human disease processes, particularly cancer and in adaptation to reproductive mode in planarians flatworms [46]. Alternative splicing has been reported in many other taxa [51–54, 89, 92, 93, 141–144]. We sought to determine whether alternative splicing of TERT is common across animals and if AS variants show any conservation. We probed available genomic and expression datasets for the presence of AS variants. We identified 19 animal species with *TERT* AS variants as evident by computational predictions using RNA-sequencing datasets (Additional file 9: Table S3; Additional file 10). We found AS variants in both protostomes (10 species) and deuterostomes (9 species). From this list, AS variants have been previously described for these five species: *Homo sapiens* [51, 56, 141, 144], *Ornithorhynchus anatinus* [93], *Gallus gallus* [54, 142], *Danio rerio* [89, 143] and *Solenopsis invicta* [92]. We observed extensive patterns of alternative splicing in hymenopteran and hemipteran insects (Additional file 11: Figure S8). Some of the most common splicing events in hymenopterans and hemipterans involved the exclusion of exon 2 (6 out of 9 species) and exon 3 (4 out of 9 species; Additional file 11: Figure S8). All 43 AS variants in insects have retained an open reading frame (ORF; Additional file 12: Figure S9). Of these, 11 have truncated TRBD (motifs CP, QFP and T) domains and nine have truncated RT (motifs 1, 2, A, B′, C, D and E) domains (Additional file 11: Figure S8). The substantial lengths and complete ORFs of many AS variants in insects indicate that they may still be functional at the protein level. In Lophotrochozoa, two AS variants were identified in the mollusc *A. californica* (Additional file 12: Figure S9). Since the combination of exons 2 and 3 only encode 39 amino acid residues, all AS variants in *A. californica* appear to retain a complete set of motifs like the full-length TERT (Additional file 12: Figure S9). Within Arthropoda, aphids and lepidopterans lack any introns and hence alternative splicing (Fig. 3). The short-lived nature of these taxa may preclude the need for highly regulated TERT activity.

Amongst vertebrate species, many AS variants have been previously described but to our knowledge, splicing patterns of *TERTs* in the Amazon molly *Poecilia formosa,* the green spotted puffer fish *Tetraodon nigroviridis*, the wild turkey *Meleagris gallopavo* and the Rhesus macaque *Macaca mulatta* have not been reported (Additional file 13: Figure S10). AS variants in *hTERT* involved exons 6 to 8 and we witness the splicing events concerning these exons in five out of nine species (3 mammals, the wild turkey and zebrafish) (Additional file 13: Figure S10), suggesting some conservation of splicing across the vertebrates. The exclusion of the unusually large exon 2 (1.3 kb) is a common 5′ splice variant in primate *TERTs* [55] and we observe this splicing event in all vertebrates except the Rhesus macaque (Additional file 13: Figure S10). The lack of observation of the AS variant in Rhesus Macaque may be due to lack of confirmatory data. Overall, many AS variants in mammals and fishes appear to be of substantial lengths, suggesting that they would encode functional proteins given that most of their TRBD and RT motifs are still present (Additional file 9: Table S3). In avians however, many AS variants appear to lack any canonical motifs. The wild turkey has 10 AS variants, eight of these involve the exclusion of multiple exons and/or loss of frame mutations, do not contain any TERT motifs and are therefore likely to be non-coding and be subjected to nonsense-mediated decay (Additional file 13: Figure S10B). This observation of complex splicing patterns generating many short AS variants with premature termination codon has also been reported in chickens [54, 142] (Additional file 13: Figure S10B). It is unknown as to whether these AS variants would participate directly or indirectly in canonical telomerase function or in other non-canonical functions of TERT. But clearly alternative splicing of the TERT protein is a pan-metazoan phenomenon. Given that we do not see broad conservation of splicing patterns (except within the vertebrate lineage) our data suggest, that in combination with the lineage specific sequence evolution we have also described, AS is likely to contribute to lineage specific functions of this core eukaryotic protein.

### Free-living planarian species exhibit complex patterns of alternative splicing

While our analysis of alternative splicing on TERT revealed extensive evidence of splicing, data available to assess evolutionary changes of splicing in taxa outside of the vertebrates is relative sparse. A previous study had investigated telomere length, telomerase activity and TERT regulation in the highly regenerative model planarian *Schmidtea mediterranea* [46]. This study found that the asexually reproducing biotype upregulated active full length splice forms of *TERT* during regeneration to sufficient levels to maintain telomere length during stem cell division. Another study in the related species *Dugesia ryukyuensis* demonstrated equivalent telomere length maintenance differences between sexual and asexual strains [145]. In order to use planarians as a study system for understanding how AS could contribute to the evolution of TERT regulation we performed a detailed investigation of *TERT* gene structure, expression and alternative splicing in Tricladida planarians, a group where most, but not all species, are known to be highly regenerative.

#### Planarian-specific motifs within the N-terminal linkers and CTE regions of TERT

**Fig. 4:**
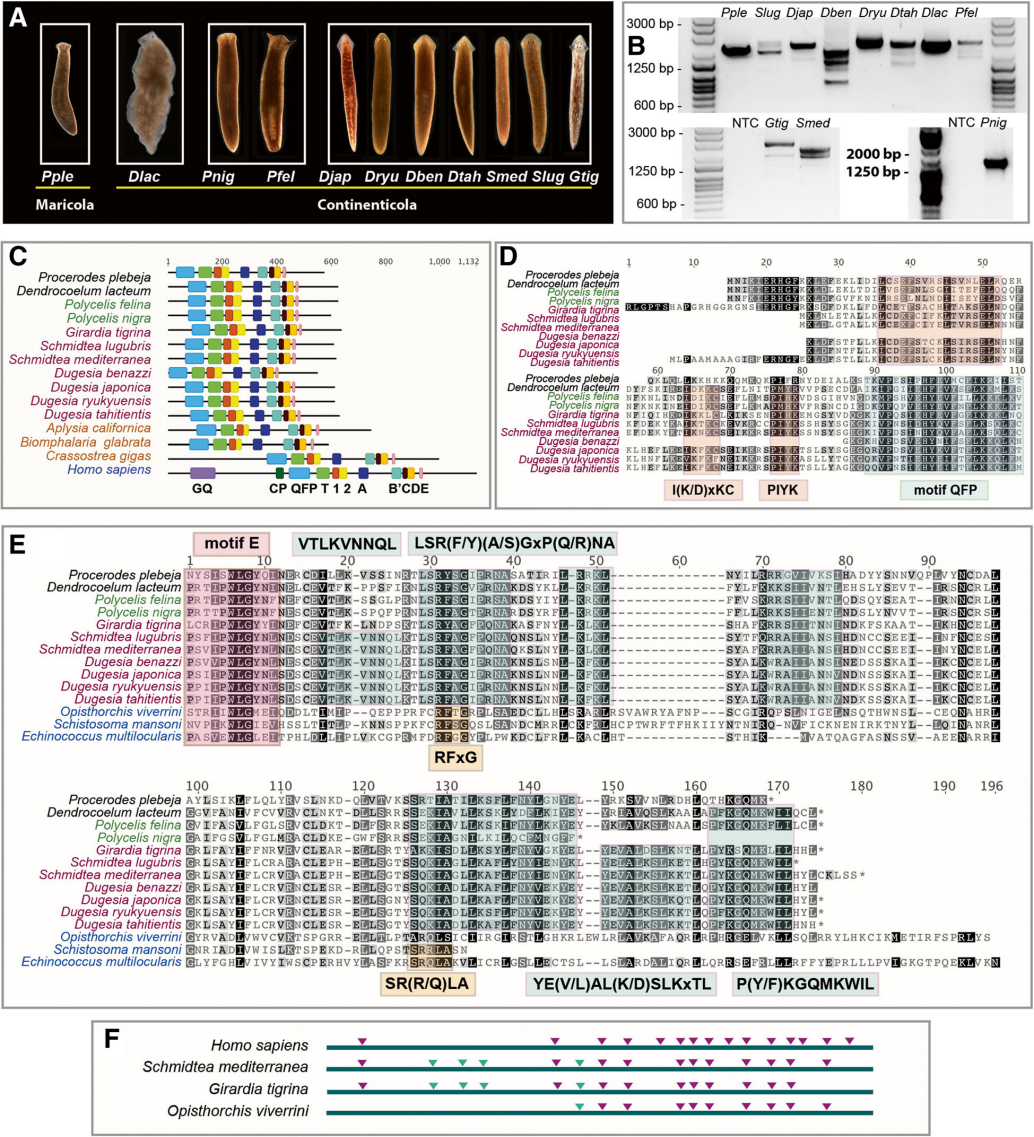
*TERT* in highly regenerative triclad planarians. **a** The 11 flatworm species used in this study. Abbreviations represent: *Pple (Procerodes plebeja), Dlac (Dendrocoelum lacteum), Pnig (Polycelis nigra), Pfel (Polycelis felina), Djap (Dugesia japonica), Dryu (Dugesia ryukyuensis), Dben (Dugesia Benazzi), Dtah (Dugesia tahitientis), Smed (Schmidtea mediterranea), Slug (Schmidtea lugubris)* and *Gtig (Girardia tigrina).* **b** Gel image showing full length RT-PCR results of *TERT* transcripts and isoforms from 11 planarian species (NTC represents no cDNA template control for the PCR). **c** Domain structure of planarian TERTs; mollusc TERTs and hTERT were used for comparison. Multiple sequence alignments of the (**d**) N-terminal linker and (**e**) C-terminal extensions from planarians and the trematode*s*. *Boxes* also denote planarian-specific motifs with their respective descriptions written inside. **f** *TERT* gene structure of *hTERT, S. mediterranea, G. tigrina* and the liver fluke *Opisthorchis viverrini* (Trematoda Class) are shown. *Purple triangles* indicate conserved intron positions (relative to *hTERT* intron positions) and *green triangles* indicate Platyhelminthes-specific introns. Genomic sequence data is not available for other planarian species. Therefore, putative *TERT* exon-intron boundaries were annotated based on *Smed_TERT* data and confirmed by AS variant analyses of each species

We obtained five new partial *TERT* sequences from published transcriptome sources or previous studies [46, 146–151]. Based on these sequences, we designed putative pan-flatworm degenerate primers and using a combination of degenerate PCR together with 5′ and 3′ RACE techniques, we cloned full-length *TERT* sequences from 11 Tricladida planarian species. These included ten Continenticola species (a clade consisting of freshwater triclads from the family Dendrocoelidae, Dugesiidae and Planariidae) and one Maricola species (a suborder of marine triclads from the family Procerodidae; Fig. 4a & b; Additional file 14: Table S4). We also confirmed both the identity of each species and phylogenetic relationships amongst taxa using 18S rDNA resequencing (Additional file 15: Figure S11; Additional file 14: Table S4) [60–62]. We observed that like other lophotrochozoans (*A. californica, Biomphalaria glabata* and *Crassostrea gigas*), TERT in triclad planarians lack the GQ and CP motifs. (Fig. 4c; Additional file 16: Figure S12). We retrieved six partial sequences for other platyhelminthes from the following classes: Trematoda (*Schistosoma mansoni, S. margrebowiei, Echinostoma caproni; Opisthorchis viverrini*) [152, 153], Cestoda (*Echinococcus multilocularis*) [154] and Turbellaria (*Macrostomum lignano*) [155]. Since these sequences are incomplete, we only used species with intact CTE or N-terminal linker regions for comparison. Although we failed to compare N-termini of parasitic flatworms, we were able to identify planarian-specific N-terminal motifs within this region such as the I(K/D)xKC and PIYK motifs (Fig. 4d). With regards to CTE regions, there is poor conservation between molluscs and platyhelminthes and they require separate alignments. Within platyhelminthes CTE regions, we observed pockets of conservation, e.g. the P(Y/F)KGQMKWIL and LSR(F/Y)(A/S)GxP(Q/R)NA motifs in triclad planarians (Fig. 4e). The VTLKVNNQL and YE(V/L)AL(K/D)SLKxTL motifs are conserved within Dugesiidae (Fig. 4e).

#### TERT intron-exon structure in planarians and the prevalence of alternative splicing

The observation of multiple PCR products when using primers that span the entire *TERT* transcript in planarians was suggestive of *TERT* alternative splicing (Fig. 4b). We wished to investigate the extent of the regulation by alternative splicing in this group of animals that exist over short evolutionary time frames. It was previously reported that *TERT* in *S. mediterranea* is alternatively spliced into four variants [46]. Analysis of *TERT* intron-exon boundaries using existing genome assembly datasets for *S. mediterranea* [149] and *Girardia tigrina* (unpublished data) revealed that *Smed-TERT* and *Gtig-TERT* have 16 and 15 exons respectively. Of these, 11 *Smed-TERT* and 10 *Gtig-TERT* introns are conserved with *hTERT* introns where they share the same positions on the TERT protein (Fig. 4f). As planarian *TERTs* shared a high degree of structural similarity and due to the lack of genomic data for the nine remaining planarian species, we based our AS variant analysis on an inferred genomic structure generated from the *Smed-TERT* sequence. From the splicing patterns of cloned AS variants, we could visualise exons that are being excluded and this confirmed that the intron-exon boundaries were accurately inferred based on *Smed-TERT* sequence (Additional file 17: Figure S13; Additional file 18: Figure S14; Additional file 19). AS variants were identified from all seven Dugesiidae species, two Planariidae species and one Dendrocoelidae species Additional file 17: Figure S13; Additional file 18: Figure S14A and B). We did not find any AS variants from the marine planarian *Procerodes plebeja* (Procerodidae), an outgroup to all other species (Additional file 18: Figure S14C). The highest number of AS variants was identified in *G. tigrina* (13 variants) with the lowest being *Dendrocoelum lacteum* (2 variants; Additional file 17: Figure S13; Additional file 18: Figure S14). Two of the shortest AS variants in *G. tigrina* did not contain any TERT motifs (Additional file 17: Figure S13). Dugesiidae species in general had more AS variants compared to Dendrocoelidae and Planariidae species. Five AS variants in *D. ryukyuensis* only have partial QFP motifs and not any other TERT motifs (Additional file 17: Figure S13). Of all 52 AS variants in triclad planarians, the most prevalent pattern is the truncation of the RT domain where 24 out of 52 have partial or missing RT domains (Additional file 17: Figure S13; Additional file 18: Figure S14; Additional file 20: Table S5). We observed 20 out of 52 AS variants containing truncated versions of both TRBD and RT domains and only 7 out of 52 contain a truncated TRBD but intact RT domain (Additional file 20: Table S5; Additional file 17: Figure S13; Additional file 18: Figure S14). Despite sharing conserved positions of introns (as inferred from splicing patterns of each species), there is low degree of conservation in the selection of spliced sites for these species. The most prevalent splicing pattern is the deletion of exons 8 to 10 (represented by orange triangles), which is present in six out of ten species where AS variants were found (Additional file 17: Figure S13; Additional file 18: Figure S14). Deletion of exons 11 and 12 (represented by blue triangles) were identified in five out of ten species. These splicing events would affect the *TERT* RT domain that is encoded by exons 8 to 13 (Additional file 17: Figure S13; Additional file 18: Figure S14). We note that the least amount of *TERT* splicing is observed in the least regenerative species, *P. plebeja* and *D. lacteum* (Additional file 21: Figure S15; Additional file 22: Figure S16) [156]. The prevalence of *TERT* splicing and regenerative capacity is broadly correlative at this stage and future functional studies will be required to ascertain whether any direct linkage between splicing and regenerative capacity exists. Overall our data suggest that AS splicing of TERT is evolving rapidly within this group of animals and that both fine grained study of *TERT* alternative splicing as well as functional study of AS variants are required to understand why *TERT* has evolved so dynamically during animal evolution.

## Conclusion

We performed thorough analyses of TERT canonical and non-canonical motifs, gene structure and alternative splicing across representative metazoan species. We show that although the ancestral TERT protein in Metazoa is likely to possess all 11 canonical motifs, the GQ and CP motifs are prone to lineage specific losses (Fig. 2). Beyond the canonical motifs, we demonstrate that the N-terminal linkers and CTE regions of TERTs are highly divergent across phyla. In these regions, we discovered novel motifs that exhibit high levels of sequence conservation over long evolutionary times indicating that they may serve an unknown but important biological function. For example, the CTE regions are distinct within phyla (Additional file 7: Figure S6) and it seems likely that such differences are reflective of life history diversity as growing evidence points to the importance of the CTE for telomerase processivity in vivo [123, 124, 157]. We also demonstrated that the N-terminal linker regions have poor sequence conservation between metazoan phyla (Additional file 8: Figure S7). The N-terminal linkers of TERT proteins have been implicated to mediate a conserved function in enhancing the translocation of the 3′ end of DNA substrate relative to the RNA template [158, 159]. Deleting residues in this region reduces the translocation of DNA substrate and overall processivity [115]. Future studies will be needed to unravel the biological significance of phyla specific sequences and whether they fine tune telomerase processivity to life history strategy.

Analysis on *TERT* intron exon structure revealed that the metazoan ancestor is intron rich and share many intron positions with *TERTs* in the vertebrate lineage (Fig. 3). *TERTs* in protostomes have undergone significant intron loss; lepidopterans and aphids do not have any introns, perhaps due to short lifespans of these taxa. Alternative splicing of TERT has been extensively studied in many species. We report new observations on splicing events for 14 metazoan species and show that the selection of spliced exons is poorly conserved in these animals (Additional file 11: Figure S8; Additional file 12: Figure S9; Additional file 13: Figure S10). Lastly, we investigated *TERT* alternative splicing in Tricladida planarians and demonstrated that splicing is dynamic and rapidly evolving within this group of closely related species (Fig. 4, Additional file 17: Figure S13; Additional file 18: Figure S14). It is likely that splicing in planarian *TERTs* is significant for the highly regenerative and potentially immortal life histories of some of these species and AS variants could possess important non-canonical functions. This study highlights that neither *TERT* structure nor its regulation is static. A future endeavour will be to unravel mechanistic linkages that connect unique sequence evolution and the dynamic regulation of TERT to actual biological roles.

## Methods

### Data mining of TERT sequences

TERT sequences were retrieved from Ensembl Genome and Ensembl Metazoa [160], NCBI and Uniprot. Sequences were confirmed using reciprocal blastx against the nr database using the NCBI Blast suite. We retrieved tardigrade TERTs from the *Hypsibius dujardini* genome browser (http://badger.bio.ed.ac.uk/H_dujardini/blast/index) and the *Ramazzottius varieornatus gene models (* http://kumamushi.org/database.html). TERT sequence from the crustacean *Parhyale hawaiensis* was retrieved from the assembled genome [97]. Additional references for other datasets are included in the main text. Complete species names, accession numbers and database sources are listed in Additional file 1: Table S2.

### Phylogenetic analyses

Multiple sequence alignment of TERT protein sequences (from motif T to motif E) was performed using MAFFT with the following parameters: scoring matrix BLOSUM62, gap penalty 1.53, offset value 0.123 [161]. Phylogenetic tree was constructed using RAxML [162] employing the WAG matrix and gamma distribution rate model with 1000 bootstrap replicates. A single tree topology was generated based on the best-scoring maximum likelihood tree. The tree Figure was made using FigTree (http://tree.bio.ed.ac.uk/software/figtree/). Branches that contained less than 30% boostrap replicates were collapsed.

### Annotation of TERT canonical motifs and non-canonical motifs in C-terminal extension (CTE) and N-terminal linker regions

TERT protein motifs (GQ, CP, QFP, T, 1, 2, A, B′, C, D and E) were annotated based on regional comparisons with hTERT protein annotations in multiple sequence alignments performed using MAFFT with the following parameters: scoring matrix BLOSUM62, gap penalty 1.53, offset value 0.123 [161]. For the alignments of canonical domains, we used published domain annotations from two reference sequences (hTERT and *C. elegans* TERT). For the alignment of TERT domains in unicellular relatives of metazoans (Additional file 3: Figure S2) and insects (Additional file 5: Figure S4), the hTERT sequence was used as a reference while for nematodes, the *C. elegans* TERT sequence was used as a reference (Additional file 4: Figure S3). Global alignments of full-length TERT sequences were first performed on the target taxa to allow the annotation of regions that align well. Regions that do not align well were extracted and local alignments were then performed with the reference sequence. For example, we identified the GQ motif in unicellular relatives of metazoans from a local alignment of the N-terminal region (Additional file 3: Figure S2). For CTE and linker regions, local alignments were performed only on selected taxa for the identification of non-canonical motifs. Fasta files for all alignments performed in this study are included (Additional files 23, 24, 25, 26, 27, 28, 29, 30, 31, 32, 33, 34, 35, 36, 37, 38, 39, 40, 41, 42, 43, 44, 45, 46, 47, 48, 49, 50, 51, 52). Alignment Figures were generated using Geneious (version 7) [163].

### Animal culture

All freshwater flatworm strains were cultured at 20 °C using the planarian water formulation. 1X Montjuic salt solution was prepared using milliQ ddH2O with the following composition: 1.6 mM NaCl, 1 mM CaCl_2_, 1 mM MgSO_4_, 0.1 mM MgCl_2_, 0.1 mM KCl, 1.2 mM NaHCO_3_ [164]. The marine flatworm *Procerodes plebeja* was cultured at 14 °C using a salt water formulation made with Tropic Marin sea salt to a salinity of 28-30 ppm. *Dendrocoelum lacteum* was fed with shrimp while all other flatworms used in this study were fed organic beef liver once a week. All flatworms were starved for 1 week prior to any experimental procedures. The worms were kept in the dark at all times apart from feeding and water changing times.

### RNA extraction and reverse transcription in Triclad flatworms

Total RNA was isolated from three animals of each species using the TRIzol reagent (Thermo Fisher Scientific) according to manufacturer’s instructions. cDNA was generated from total RNA extract using the QuantiTect reverse transcription kit (Qiagen) containing the RT primer mix (blend of oligo-dT and random hexamers). A genomic DNA elimination step was also included in this kit performed using the gDNA wipeout buffer.

### Degenerate polymerase chain reaction (PCR) and 5′ 3′ RACE for the cloning of *TERT* genes

*TERT* transcript sequences for *Girardia tigrina* [148]*, Dugesia japonica* [147]*, D. lacteum,* and *P. nigra* [151] were retrieved using tblastn with *Smed_TERT* as a query from published transcriptome sequences of the respective species. Top hits with the best e-value for each species was used for reciprocal blast against the nr database in NCBI to confirm the identification of *TERT.* For degenerate PCR, first strand cDNA synthesis was performed as mentioned previously. For 5′ and 3′ RACE, first strand cDNA synthesis was performed on TRIzol extracted total RNA using the SMARTer RACE 5′ 3′ Kit (Clontech).

For the cloning of *TERT* in all the other *Dugesia sp.* (*Dugesia ryukyuensis, Dugesia tahitientis, Dugesia benazzi*) degenerate PCR primers were designed based on the pairwise alignment of *Smed-TERT* and *Djap-TERT*: DugesiaFD (5′-CACTGGTGYGAATCACCA-3′) and DugesiaRD (5′- AAAACATCATCMACRTATTG-3′). PCR products were cloned in the pGEMT-easy vector (Promega) followed by colony PCR with M13F (5′- GTAAAACGACGGCCAGT-3′) and M13R (5′- GGAAACAGCTATGACCATG-3′) primers. Positive colonies were selected for Sanger sequencing. Once the middle sequence of *TERT* transcripts for all three *Dugesia sp.* were obtained, 5′ and 3′ RACE was performed on cDNA made with the SMARTer RACE 5′ 3′ Kit using gene specific primers listed in Additional file 14: Table S4 according to manufacturer’s instructions.

For the cloning of *TERT* in *Schmidtea lugubris*, the same set of degenerate PCR primers (DugesiaFD and DugesiaRD) was used for PCR followed by cloning and sequencing. PCR products were cloned in the pGEMT-easy vector (Promega) followed by colony PCR and sequencing as mentioned previously. The 5′ end of *Slug_TERT* transcript was cloned using the following primer set: Dugesia5F (5′-AATYGAGMGWMATGGTTT-3′; this primer was designed based on 5′ sequence region in *Dugesia sp.*) and LugR (5′- CTGAAATTTGTGCCATTG-3′; *Slug-TERT* gene specific primer). Next, 3′ RACE was performed with the SMARTer RACE 5′ 3′ Kit using gene specific primers listed in Additional file 14: Table S4 according to manufacturer’s instructions.

For the cloning of *TERT* in *Polycelis felina*, degenerate PCR primers were designed based on pairwise alignment of *Pnig-TERT* and *Polycelis tenuis TERT* (obtained from PlanMine) [151]: PolycelisDF (5′-AATTGGCACMTSTTYCTG-3′) and PolycelisDR (5′-GACTCRTARCAAYTCTT-3′). PCR products were cloned in the pGEMT-easy vector (Promega) followed by colony PCR and sequencing as mentioned previously. The 5′ and 3′ end of *Pfel-TERT* was obtained using 5′ and 3′ RACE with the SMARTer RACE 5′ 3′ Kit using gene specific primers listed in Additional file 14: Table S4 according to manufacturer’s instructions.

For the cloning of *TERT* in *P. plebeja*, degenerate PCR primers were designed based on pairwise alignment of *Pnig_TERT* and *Pfel_TERT*: PpleDF (5′- ATGSARTATAAAGGATWTAT-3′) and PpleDR (5′-AAYRTCRTCGACATATTG-3′). PCR products were cloned in the pGEMT-easy vector (Promega) followed by colony PCR and sequencing as mentioned previously. The 5′ and 3′ end of *Pple-TERT* was obtained using 5′ and 3′ RACE with the SMARTer RACE 5′ 3′ Kit using gene specific primers listed in Additional file 14: Table S4 according to manufacturer’s instructions. All degenerate PCRs were performed using the Advantage 2 Polymerase (Clontech) according to recommended thermal cycling parameters. All RACE products were ran on a gel and gel extraction was performed with the MinElute gel extraction kit (Qiagen) following by cloning and sequencing of the RACE products.

### DNA extraction, 18S rDNA PCR and sequencing

High molecular weight genomic DNA (gDNA) was isolated using the phenol-chloroform method followed by ethanol precipitation. PCR was performed according to conditions in Carranza et al. [61] using the Advantage 2 Polymerase (Clontech). List of 18S primer sequences are provided in Additional file 14: Table S4. PCR products were gel extracted using the MinElute gel extraction kit (Qiagen) and eluted in 10uL of molecular grade water. Gel extracted products were sequenced in both directions using 18S nested primers listed in Additional file 14: Table S4. Sequences were aligned with known 18S sequence data available from NCBI (accession numbers provided in Additional file 14: Table S4). 18S rDNA sequences obtained from this study fell into two groups (18S type I and 18S type II).

### Cloning of planarian *TERT* AS variants

PCR primers were designed to amplify from the start to end of *TERT* transcript sequences obtained from above. Full list of primers used to amplify *TERT* AS variants are provided in Additional file 14: Table S4. PCR was performed using Phusion Polymerase (Thermo Fisher Scientific) and all PCR products were ran on a gel. In order to cut out gel pieces containing the AS variants, the gel image was overexposed to enable visualization of low abundance variants. Gel regions containing bands were excised and extracted using the MinElute gel extraction kit (Qiagen). A-tailing was subsequently performed with GoTaq Polymerase (Promega) for TA-cloning purposes since Phusion PCR products were blunt ended. PCR products were cloned in the pGEMT-easy vector (Promega) followed by colony PCR with M13F and M13R primers. Colony PCR products were run on a gel for 2 h using 1.3% agarose in 1X TAE buffer to allow good resolution of bands. Colony PCR gel would display an array of bands with different sizes representing the AS variants. Bands of different sizes were selected for Sanger sequencing. For each species, 25 colonies were selected for sequencing to allow exhaustive identification of *TERT* AS variants.

### Sequence analyses of flatworm *TERT* AS variants

Eleven planarian species were used in this study. Planarians *Schmidtea mediterranea, S. lugubris, G. tigrina, D. japonica, D. benazzi, D. tahitientis, P. felina*, *P. nigra* and *D. lacteum* were purchased from Sciento (http://www.sciento.co.uk/). *D. ryukyuensis* was a gift from Dr. *Midori Matsumoto (Keio University, Japan) and P. plebeja* from Dr. Bernhard Egger (Universität *Innsbruck)*. The gene structures (exon-intron boundaries) for *Smed-TERT* and *Gtig-TERT* were identified using blastn against the *S. mediterranea* [149] and *G. tigrina* genome assembly (unpublished data). Both *Smed-TERT* and *Gtig-TERT* have almost identical intron positions, with *Gtig-TERT* having one less exon. Since genome data is not available for all the other flatworm species, we based our analyses on the gene structure of *Smed-TERT* and *Gtig-TERT* and refer to them as inferred exon positions. Further analyses on cloned AS variants for species without genomic data confirmed the accurate positioning of intron-exon boundaries based on the excluded exons. The full-length *TERT* sequences for each species were referred to as ‘isoform 1’. Cloned AS variants were mapped to ‘isoform 1’, which set as a reference sequence for each respective species. From this, we were able to visualize the skipped exons, splice site mutations or retained introns by comparing the AS variant sequences to the reference sequence. All the AS variants including the longest isoform were in silico translated using Geneious (version 7) [163] to the correct open reading frame. Multiple sequence alignments of all AS protein sequences for each respective species were performed using MAFFT with the following parameters: scoring matrix BLOSUM62, gap penalty 1.53, offset value 0.123 [161]. Annotations of TERT canonical motifs were performed according to positions in hTERT; telomerase RNA-binding domain motifs (QFP and T) and canonical reverse transcriptase motifs (1, 2, A, B′, C, D and E) in all AS protein variants. Further information on the flatworm AS variants were provided in Additional file 20: Table S5.

### Annotation of *TERT* AS variants in vertebrates, insects and mollusc

The annotation of *TERT* AS variants were performed based on publically available transcriptomic and genomic datasets (Additional file 1: Table S2). To evaluate alternatively spliced exons, we have identified 19 species that contained more than one *TERT* transcripts in the transcriptomes and these additional *TERT* transcripts were considered as *TERT* AS variants. These *TERT* transcripts were in silico translated according to the correct open reading frame into protein sequences. Subsequent analyses were performed as described in the previous section “Sequence analyses of flatworm *TERT* AS variants”. The longest transcripts/isoforms were used as reference sequences and all other AS variants were mapped to the reference using Geneious (version 7) [163]. This allowed the visualization of skipped exons, splice site mutations or retained introns by comparing the AS variant sequences to the reference sequence. Detailed descriptions on splicing patterns are available in Additional file 9: Table S3.

### Annotation of intron positions on TERT protein sequences

Methods for the identification of conserved intron positions were adapted from two previous reports [139, 165]. The locations of the exon boundaries were first annotated on TERT transcripts based on their cognate genomic sequences (schematic diagram in Additional file 53: Figure S17). Next, the transcripts were in silico translated to protein sequences while retaining information on the location of each exon boundary (also referred here as intron positions). TERT protein sequences were independently aligned with the hTERT sequence. Pairwise alignments with hTERT were performed to allow the generation of a schematic diagram that is scaled according to the hTERT sequence. From the pairwise alignments, intron positions that overlap with hTERT introns were defined as conserved introns, while intron positions that do not overlap with hTERT introns were defined as species-specific introns (Additional file 53: Figure S17).

## Additional files

Additional file 1: Table S2. TERT sequences from representative species and their corresponding accession numbers. (XLSX 57 kb)

Additional file 2: Figure S1. Graphs showing sequence conservation of TERT canonical motifs: TEN domain (GQ), TRBD domain (CP, QFP and T), and RT domain (1, 2, A, B′, C, D and E). The y-axes represent frequency of the most-abundant amino acid residue at any given position with a frequency of 1.0 indicating 100%. The x-axes represent the most-abundant amino acid residue at any given position across the length of the motif. (PDF 115 kb)

Additional file 3: Figure S2. Multiple sequence alignments of the (A) TEN domain (GQ motif) and TRBD domain (CP, QFP, T) and (B) RT domain (1, 2, A, B′, C, D and E) from unicellular relatives of metazoans. Areas highlighted in coloured boxes delimit the canonical motifs. Boxes also denote species- or class-specific motifs with their respective descriptions written inside. Except for *Capsaspora,* the protozoan orders are represented by colour codes in the figure inset. (PDF 2723 kb)

Additional file 4: Figure S3. Multiple sequence alignments of the (A) GQ and CP motifs and (B) T motif and RT domain (1, 2, A, B′, C, D and E) from nematodes. Areas highlighted in coloured boxes delimit the canonical motifs. Boxes also denote Species- or Class-specific motifs with their respective descriptions written inside. Nematode classes are represented by colour codes in the figure inset. (PDF 2291 kb)

Additional file 5: Figure S4. Multiple sequence alignments of the (A) TRBD domain (CP, QFP and T) and (B) and RT domain (1, 2, A, B′, C, D and E) from insects. Areas highlighted in coloured boxes delimit the canonical motifs. Boxes also denote Species- or order-specific motifs with their respective descriptions written inside. Insect orders are represented by colour codes in the figure inset. (PDF 3367 kb)

Additional file 6: Table S1. Analyses of functional divergence between gene clusters based on model-free and maximum likelihood estimates. (XLSX 31 kb)

Additional file 7: Figure S6. Multiple sequence alignments of the C-terminal extensions (CTEs) from (A) early-branching metazoans, (B) molluscs, (C) unicellular relatives of metazoans, (D) insects, (E) nematodes, (F) crustaceans and (G) vertebrates. The last TERT canonical motif E is highlighted in pink boxes to illustrate the start of CTE regions. Boxes also denote phylum-, order- or class-specific motifs with their respective descriptions written inside. (PDF 6781 kb)

Additional file 8: Figure S7. Multiple sequence alignments of the N-terminal linker regions from (A) early-branching metazoans, (B) crustaceans, (C) unicellular relatives of metazoans, (D) insects, (E) molluscs and (F) vertebrates. Boxes denote phylum-, order- or class-specific motifs with their respective descriptions written inside. (PDF 2999 kb)

Additional file 9: Table S3. Alternatively spliced variants from vertebrates, insects and mollusc. (XLSX 49 kb)

Additional file 10: Fasta file of TERT alternatively spliced variants from representative metazoan species. (FASTA 184 kb)

Additional file 11: Figure S8. *TERT* alternative splicing in Hymenopteran and Hemipteran insects. *TERT* AS variants data were obtained from Ensembl Genomes or NCBI. These are generated by computational predictions based on RNA sequencing read evidence for (A) hymenopterans and (B) hemipterans. Schematic diagram depicts gene structure and splicing of *TERT*. Orange boxes represent exons conserved with *hTERT*. Blue boxes represent Hymenoptera specific exons while green boxes represent Hemiptera specific exons. Gray boxes represent skipped or deleted exons resulted from alternative splicing events. ‘M’ denotes splice site mutations, deletions or intron retention. The left margin shows *TERT* gene and AS variant names for each species and the right margin shows descriptive names of *TERT* AS sequences. The numbers in parentheses represent the length of splice site mutations (deletion or intron retention) for the respective AS variants. Schematic diagrams on the far right illustrate the presence or absence of canonical motifs (CP, QFP, T, 1, 2, A, B′, C, D and E) on TERT AS protein variants drawn to scale. (PDF 774 kb)

Additional file 12: Figure S9. *TERT* alternative splicing in mollusc. Schematic diagram depicts gene structure and *TERT* AS variants. Yellow boxes represent *Aplysia californica* exons. Gray boxes represent skipped exons resulted from alternative splicing events. ‘M’ denotes splice site mutations, deletions or intron retention. The left margin shows *TERT* gene and AS variant names for each species and the right margin shows descriptive names of *TERT* AS sequences. The numbers in parentheses represent the length of splice site mutations (deletion or intron retention) for the respective AS variants. Schematic diagrams on the far right illustrate the presence or absence of canonical motifs (QFP, T, 1, 2, A, B′, C, D and E) on TERT AS protein variants drawn to scale. (PDF 178 kb)

Additional file 13: Figure S10. *TERT* alternative splicing in vertebrates. AS variants are shown for (A) mammals, (B) birds and (C) fishes. Only the main AS variants for *hTERT* and chicken *TERT* are shown in this diagram. Additional variants have been reported in Hrdličková et al. [93]. The presence of AS variants with skipped exon 2 is conserved across different vertebrate species*.* AS variants with spliced exons 7 to 8 are conserved in mammals except in *G. gorilla gorilla.* Mammalian *TERT* has 16 exons whereas *TERTs* in non-mammalian vertebrates have anywhere between 14 to 17 exons. *TERT* AS variants data was obtained from Ensembl Genomes or NCBI. These are generated by computational predictions based on RNA sequencing read evidence. Schematic diagram depicts gene structure and splicing of *TERT*. Orange boxes represent exons conserved with *hTERT*. Purple and green boxes represent species-specific exons. Grey boxes represent skipped or deleted exons resulted from alternative splicing events. ‘M’ denotes splice site mutations, deletions or intron retention. The left margin shows *TERT* gene and AS variant names for each species and the right margin shows descriptive names of *TERT* AS sequences. The numbers in parentheses represent the length of splice site mutations (deletion or intron retention) for the respective AS variants except for platypus (*O. anatinus*) and chicken (*G. gallus*). For these two species, the numbers in parentheses represent the position of deletion in the exon indicated. Schematic diagrams on the far right illustrate the presence or absence of canonical motifs (GQ, CP, QFP, T, 1, 2, A, B′, C, D and E) on TERT AS protein variants drawn to scale. (PDF 334 kb)

Additional file 14: Table S4. Primers for 18S rDNA PCR and sequencing, 5′ and 3′ RACE and amplification of TERT alternatively spliced variants in triclad planarians. (XLSX 53 kb)

Additional file 15: Figure S11. Phylogenetic tree illustrating the positions of planarian type I and type II sequences in the 18S rDNA phylogeny. Sequences of 18S rDNA were cloned from 11 flatworm species in the lab for comparison against published sequences in GenBank. (A) Type I 18S rDNA sequences with species marked in blue as cloned in the lab. (B) Type II 18S rDNA sequences with species marked in brown as cloned in the lab. Asterisks indicate novel 18S rDNA sequences obtained from this study. (PDF 139 kb)

Additional file 16: Figure S12. Alignment of canonical motifs of TERT from 11 planarian species, three molluscs and hTERT. These include TRBD domain (QFP and T motifs) and canonical reverse transcriptase motifs (1, 2, A, B′, C, D and E). Clearly defined GQ and CP motifs were not identified from planarian TERTs. (PDF 1901 kb)

Additional file 17: Figure S13. *TERT* in Dugesiidae is alternatively spliced. Comparison of alternatively spliced (AS) variants in seven Dugesiidae species from the genera *Schmidtea, Girardia and Dugesia.* Structure of AS variants in *S. mediterranea, S. lugubris, G. tigrina, D. japonica, D. ryukyuensis, D. benazzi and D. tahitientis.* Full-length or wild-type *TERT* structure is shown at the top of each set of AS variants. Functional TERT domains (TRBD and RT) and positions of (putative) exons are indicated. Deletions (skipped exons) are denoted by triangles and insertions (retained introns or splice site mutations) are denoted by grey rectangles. ‘M’ abbreviations represent splice site mutations. Red triangles represent conserved alternatively spliced exons in all Dugesiidae species except *S. mediterranea* and *D. tahitientis.* Blue triangles represent conserved alternatively spliced exons in all Dugesiidae species except *G. tigrina* and *D. japonica.* Asterisks indicate stop codon positions caused by frame shift mutation or retained introns. The left margin shows *TERT* gene and AS variant names for each species and the right margin shows descriptive names of *TERT* AS sequences. Schematic diagrams on the far right illustrate the presence or absence of canonical motifs (QFP, T, 1, 2, A, B′, C, D and E) on TERT AS protein variants drawn to scale. (JPEG 1654 kb)

Additional file 18: Figure S14. *TERT* in Planariidae and Dendrocoelidae but not Procerodidae is alternatively spliced. Comparison of AS variants in two Planariidae species from the genus *Polycelis*, one Dendrocoelidae species and one Procerodidae species. Structure of AS variants in (A) Planariidae *P. felina* and *P. nigra,* (B) Dendrocoelidae *D. lacteum* and (C) Procerodidae *P. plebeja.* Genomic sequence data is not available for these flatworm species. Therefore, putative *TERT* exon-intron boundaries were annotated based on *Smed_TERT* sequence and confirmed by AS variant analyses of each species. Full-length or wild-type *TERT* structure is shown at the top of each set of AS variants. Functional TERT domains (TRBD and RT) and positions of (putative) exons are indicated. Deletions (skipped exons) are denoted by triangles and insertions (retained introns or splice site mutations) are denoted by gray rectangles. Red triangles represent alternatively spliced exons conserved with Dugesiidae. Asterisks indicate stop codon positions caused by frame shift mutation or retained introns. The left margin shows *TERT* gene and AS variant names for each species and the right margin shows descriptive names of *TERT* AS sequences. Schematic diagrams on the far right illustrate the presence or absence of canonical motifs (QFP, T, 1, 2, A, B′, C, D and E) on TERT AS protein variants drawn to scale. (JPEG 673 kb)

Additional file 19: Fasta file of planarian TERT alternatively spliced variants. (FASTA 85 kb)

Additional file 20: Table S5. TERT alternatively spliced variants and respective features in triclad planarians. (XLSX 47 kb)

Additional file 21: Figure S15. Regenerative capacity in planarians. Transverse sections assaying regenerative abilities along anterior-posterior axis of planarians. Ten worms were used for each species and were amputated into three fragments each (head, trunk and tail). Representative images depict time-course observation of the same animal undergoing cephalic regeneration in (A) trunk and (B) tail fragments. Anterior blastema (unpigmented region) was imaged. Appearances of pigmented eyes were noted and plotted as frequency graphs. Scale bars indicate 0.5 mm. (JPEG 3354 kb)

Additional file 22: Figure S16. Head regeneration frequency graph for (A) trunk fragments and (B) tail fragments of amputated flatworms (*N* = 10 for each species). Cartoon depicts the amputation planes used. The timing of eye appearance in cohorts of 10 fragments was noted. *P. plebeja* failed to regenerate at all and is not shown. (PDF 182 kb)

Additional file 23: Multiple sequence alignment of the DAT and GQ motifs in unicellular relatives of metazoans. (FASTA 737 bytes)

Additional file 24: Multiple sequence alignment of the CP motif in unicellular relatives of metazoans. (FASTA 264 bytes)

Additional file 25: Multiple sequence alignment of the QFP and T motifs in unicellular relatives of metazoans. (FASTA 4 kb)

Additional file 26: Multiple sequence alignment of the motifs 1 and 2 in unicellular relatives of metazoans. (FASTA 2 kb)

Additional file 27: Multiple sequence alignment of the A motif in unicellular relatives of metazoans. (FASTA 998 bytes)

Additional file 28: Multiple sequence alignment of the B′ motif in unicellular relatives of metazoans. (FASTA 1 kb)

Additional file 29: Multiple sequence alignment of the C and D motifs in unicellular relatives of metazoans. (FASTA 1 kb)

Additional file 30: Multiple sequence alignment of the E motif in unicellular relatives of metazoans. (FASTA 641 bytes)

Additional file 31: Multiple sequence alignment of the DAT, GQ and CP motifs in nematodes. (FASTA 2 kb)

Additional file 32: Multiple sequence alignment of the T, 1, 2, A, B′, C, D and E motifs in nematodes. (FASTA 4 kb)

Additional file 33: Multiple sequence alignment of the CP motif in insects. (FASTA 620 bytes)

Additional file 34: Multiple sequence alignment of the QFP and T motifs in insects. (FASTA 3 kb)

Additional file 35: Multiple sequence alignment of the 1, 2, A, B′, C, D and E motifs in insects. (FASTA 6 kb)

Additional file 36: Multiple sequence alignment of the CTE region in early branching metazoans. (FASTA 308 bytes)

Additional file 37: Multiple sequence alignment of the CTE region in molluscs. (FASTA 925 bytes)

Additional file 38: Multiple sequence alignment of the CTE region in unicellular relatives of metazoans. (FASTA 6 kb)

Additional file 39: Multiple sequence alignment of the CTE region in insects. (FASTA 5 kb)

Additional file 40: Multiple sequence alignment of the CTE region in nematodes. (FASTA 1 kb)

Additional file 41: Multiple sequence alignment of the CTE region in crustaceans. (FASTA 1 kb)

Additional file 42: Multiple sequence alignment of the CTE region in deuterostomes. (FASTA 8 kb)

Additional file 43: Multiple sequence alignment of the N-terminal linker region in early branching metazoans. (FASTA 627 bytes)

Additional file 44: Multiple sequence alignment of the N-terminal linker region in crustaceans. (FASTA 714 bytes)

Additional file 45: Multiple sequence alignment of the N-terminal linker region in kinetoplastids. (FASTA 1 kb)

Additional file 46: Multiple sequence alignment of the N-terminal linker region in *C. owczarzaki*. (FASTA 313 bytes)

Additional file 47: Multiple sequence alignment of the N-terminal linker region in piroplasmids. (FASTA 455 bytes)

Additional file 48: Multiple sequence alignment of the N-terminal linker region in ants. (FASTA 1019 bytes)

Additional file 49: Multiple sequence alignment of the N-terminal linker region in bees. (FASTA 954 bytes)

Additional file 50: Multiple sequence alignment of the N-terminal linker region in lepidopterans. (FASTA 345 bytes)

Additional file 51: Multiple sequence alignment of the N-terminal linker region in molluscs. (FASTA 159 bytes)

Additional file 52: Multiple sequence alignment of the N-terminal linker region in vertebrates. (FASTA 3 kb)

Additional file 53: Figure S17. Schematic diagram demonstrating the annotation of intron positions in pairwise alignments with the hTERT sequence as a reference. Amino acid residues are highlighted in black (amino acids that are conserved amongst all sequences), grey (amino acids with similar properties) and light grey (amino acids that are dissimilar). (PDF 134 kb)
