# Supplementary figures and images for: The protein subunit of telomerase displays patterns of dynamic evolution and conservation across different metazoan taxa
Source: BMC Evol Biol. 2017 Apr 26;17:107. doi: 10.1186/s12862-017-0949-4 (PMC5405514; doi:10.1186/s12862-017-0949-4)

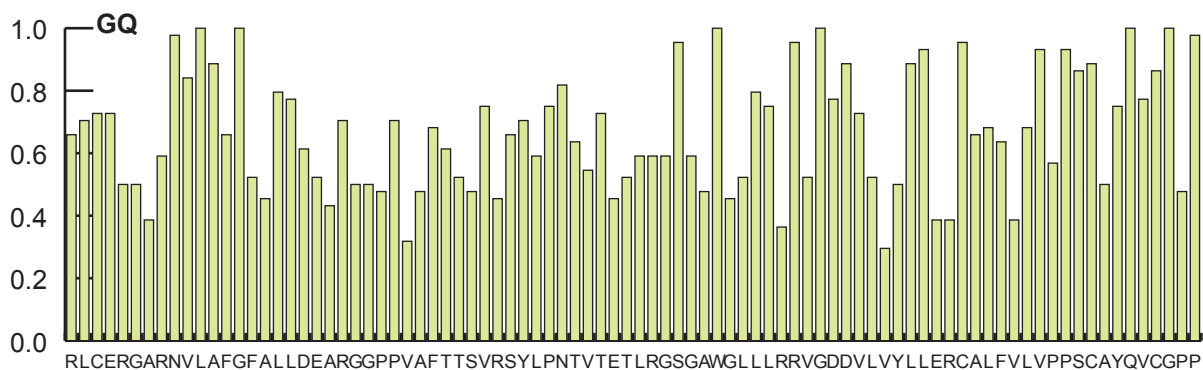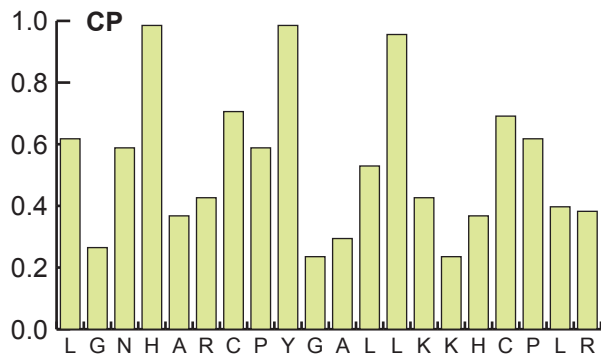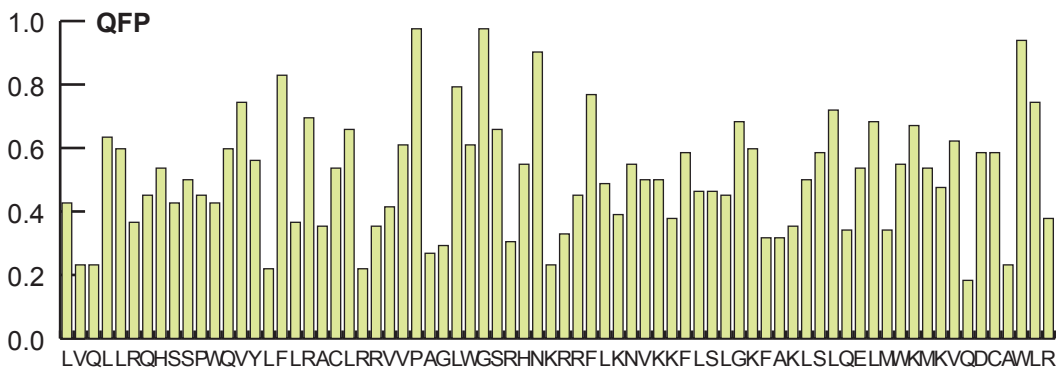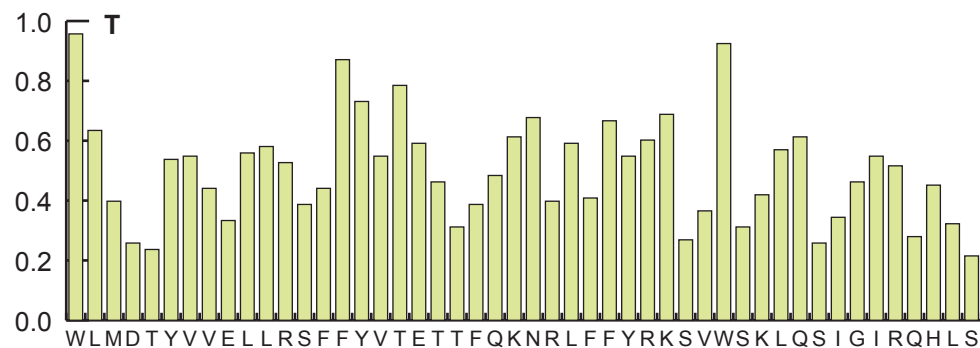

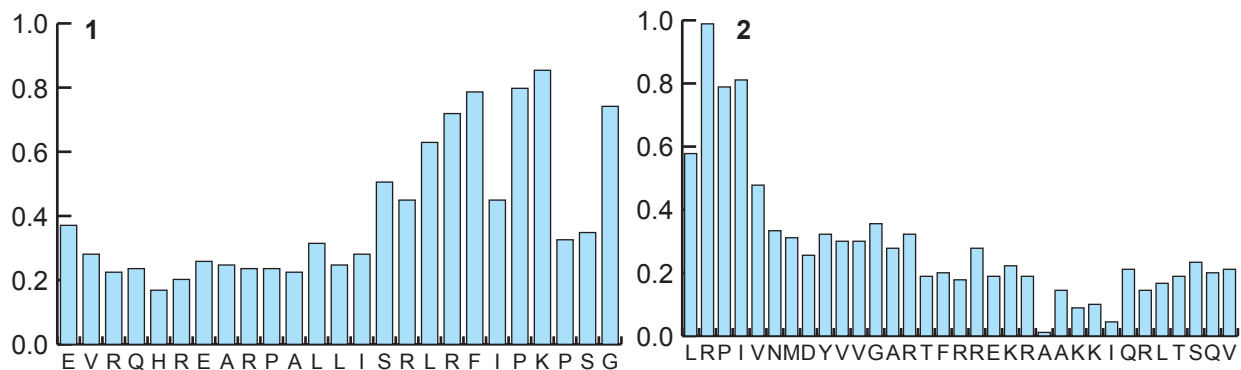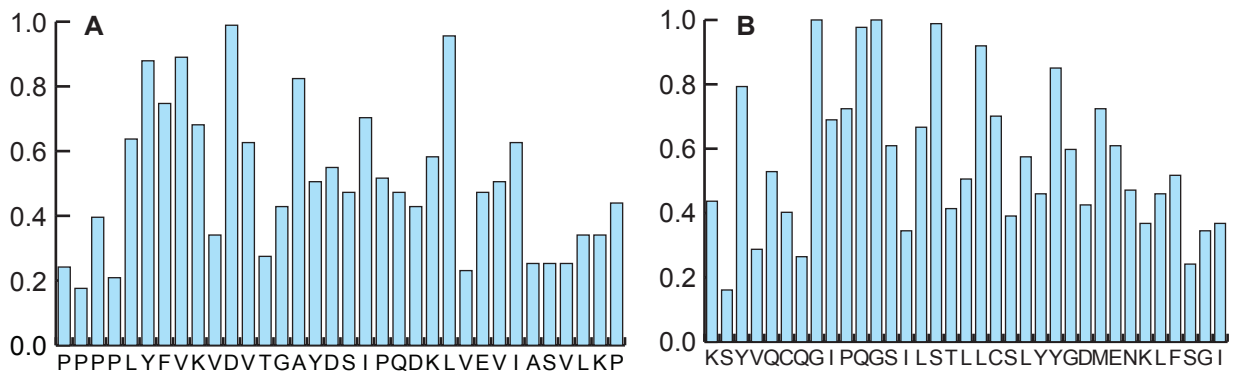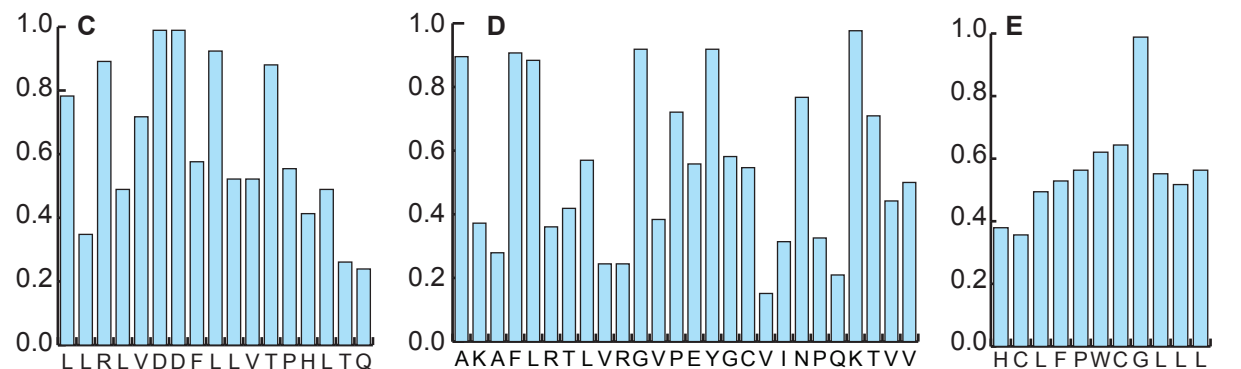

Supplement: Supplementary file 2 — Graphs showing sequence conservation of TERT canonical motifs: TEN domain (GQ), TRBD domain (CP, QFP and T), and RT domain (1, 2, A, B′, C, D and E). The y-axes represent frequency of the most-abundant amino acid residue at any given position with a frequency of 1.0 indicating 100%. The x-axes represent the most-abundant amino acid residue at any given position across the length of the motif. (PDF 115 kb) [file 12862_2017_949_MOESM2_ESM.pdf]

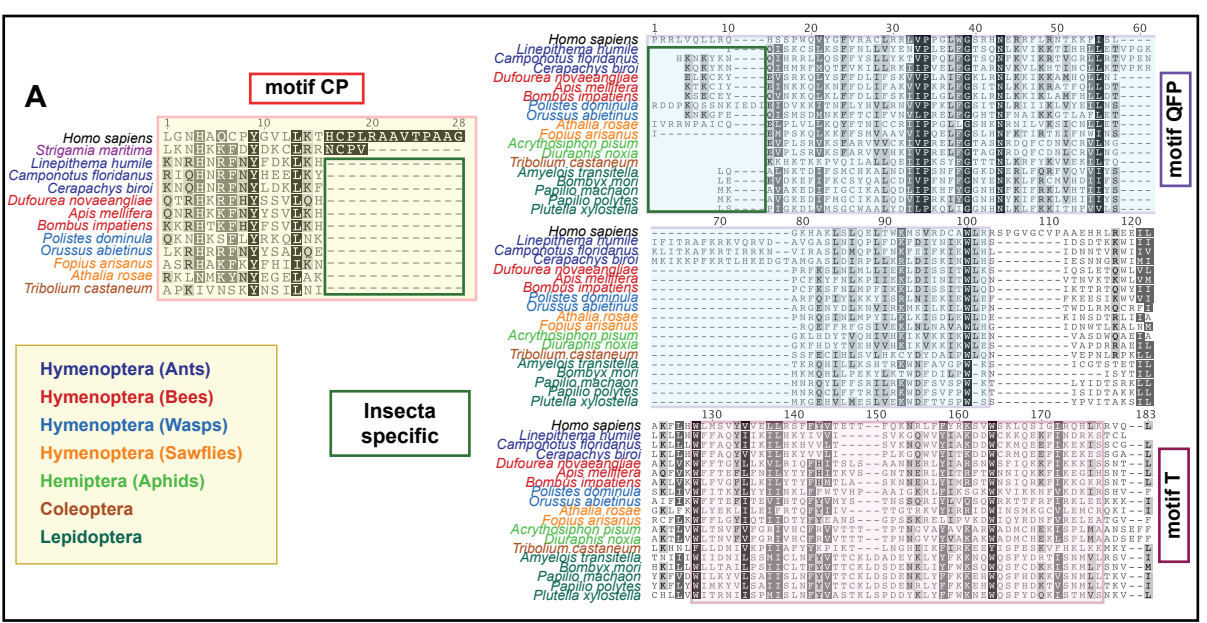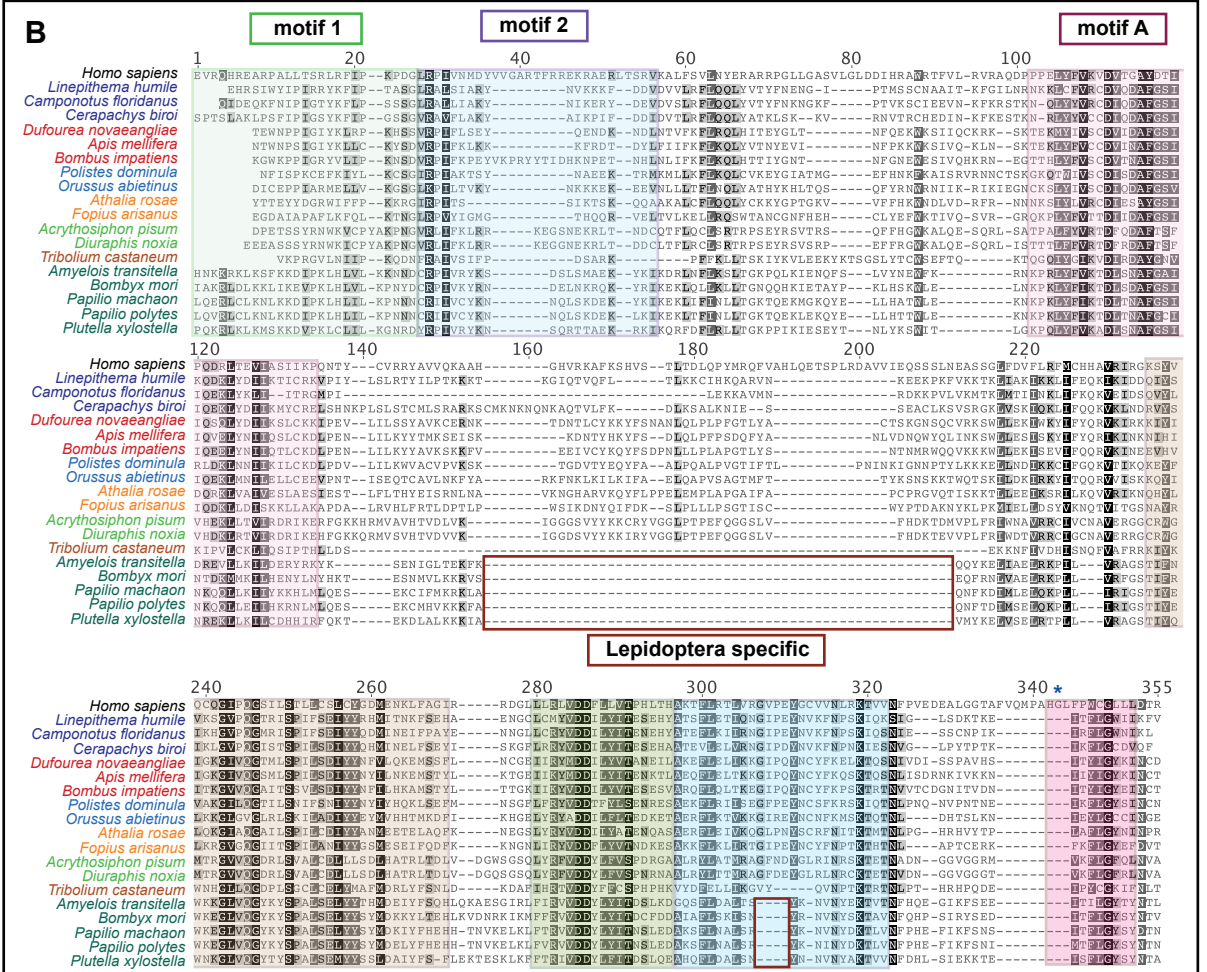

Supplement: Supplementary file 5 — Multiple sequence alignments of the (A) TRBD domain (CP, QFP and T) and (B) and RT domain (1, 2, A, B′, C, D and E) from insects. Areas highlighted in coloured boxes delimit the canonical motifs. Boxes also denote Species- or order-specific motifs with their respective descriptions written inside. Insect orders are represented by colour codes in the figure inset. (PDF 3367 kb) [file 12862_2017_949_MOESM5_ESM.pdf]

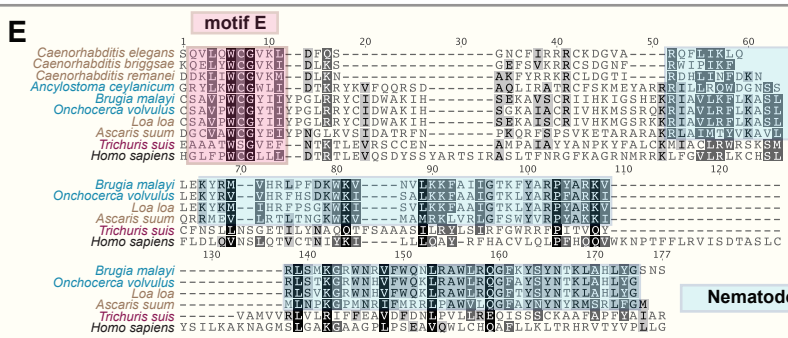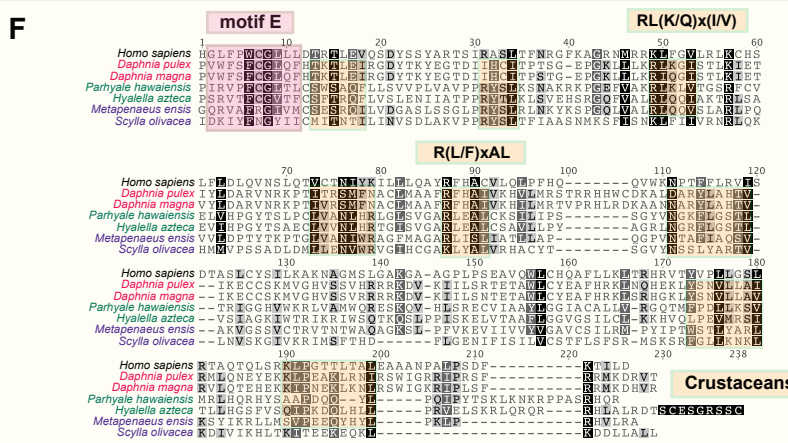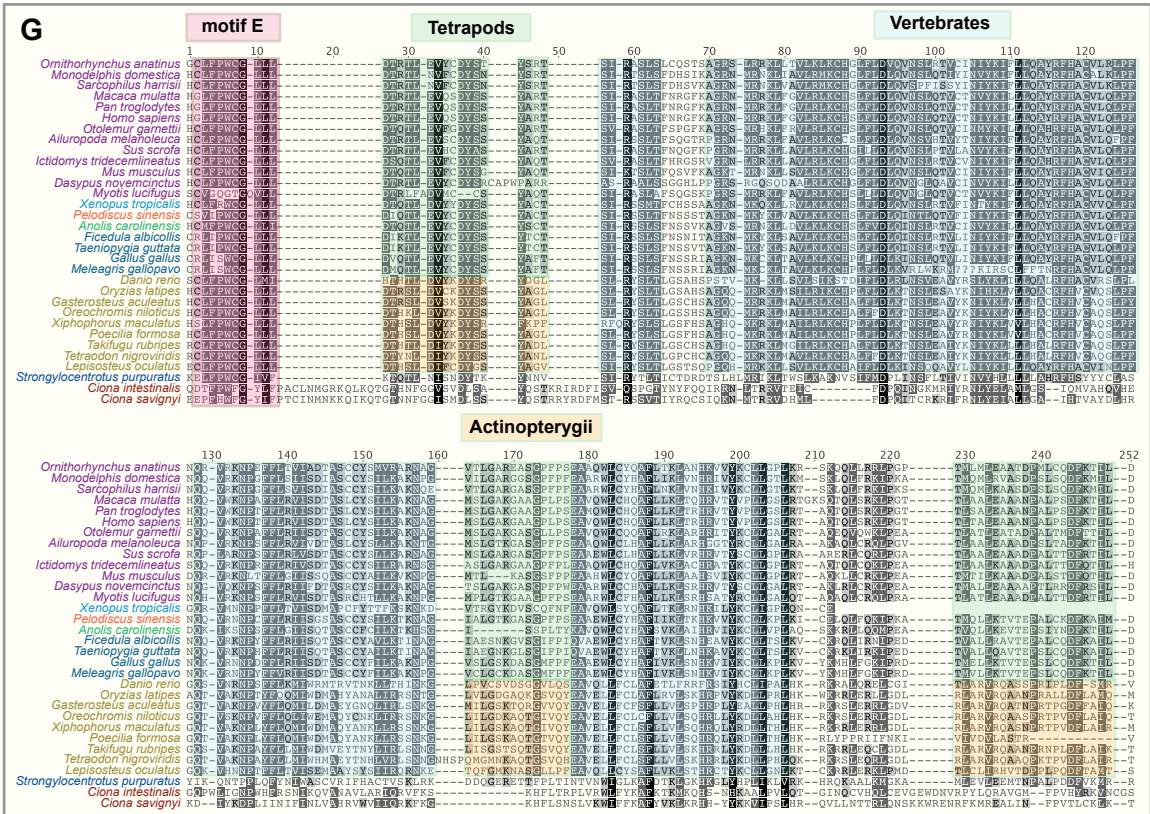

Supplement: Supplementary file 7 — Multiple sequence alignments of the C-terminal extensions (CTEs) from (A) early-branching metazoans, (B) molluscs, (C) unicellular relatives of metazoans, (D) insects, (E) nematodes, (F) crustaceans and (G) vertebrates. The last TERT canonical motif E is highlighted in pink boxes to illustrate the start of CTE regions. Boxes also denote phylum-, order- or class-specific motifs with their respective descriptions written inside. (PDF 6781 kb) [file 12862_2017_949_MOESM7_ESM.pdf]

**A**

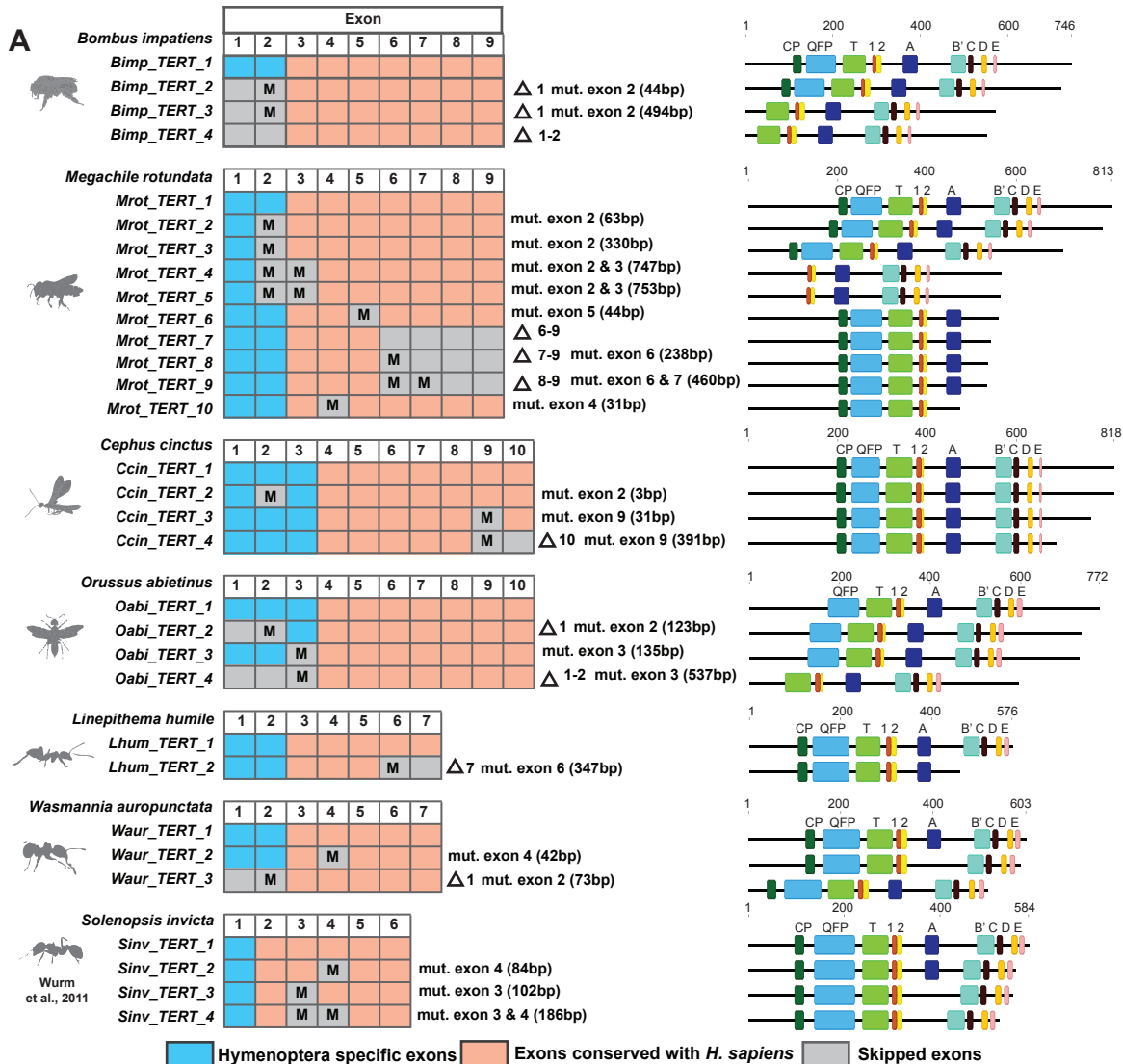

**B**

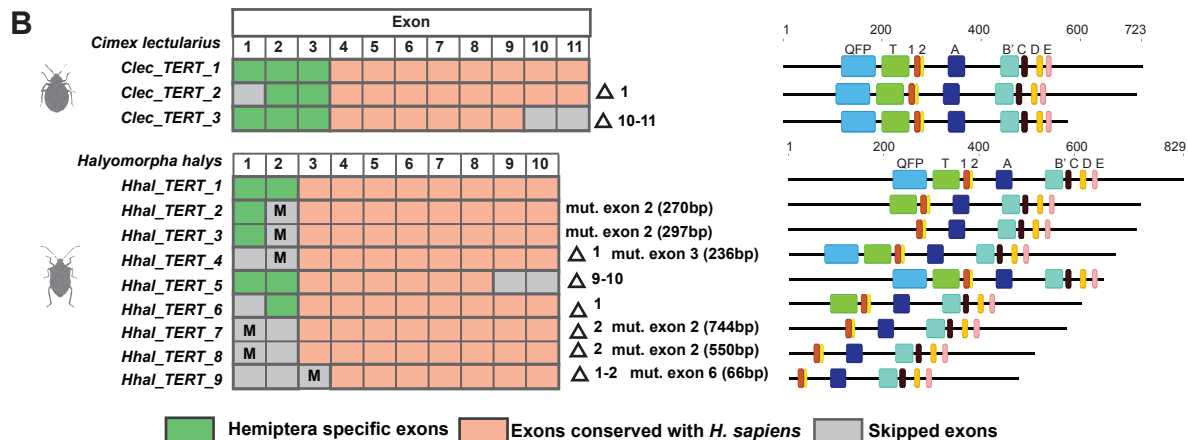

Supplement: Supplementary file 11 — TERT alternative splicing in Hymenopteran and Hemipteran insects. TERT AS variants data were obtained from Ensembl Genomes or NCBI. These are generated by computational predictions based on RNA sequencing read evidence for (A) hymenopterans and (B) hemipterans. Schematic diagram depicts gene structure and splicing of TERT. Orange boxes represent exons conserved with hTERT. Blue boxes represent Hymenoptera specific exons while green boxes represent Hemiptera specific exons. Gray boxes represent skipped or deleted exons resulted from alternative splicing events. ‘M’ denotes splice site mutations, deletions or intron retention. The left margin shows TERT gene and AS variant names for each species and the right margin shows descriptive names of TERT AS sequences. The numbers in parentheses represent the length of splice site mutations (deletion or intron retention) for the respective AS variants. Schematic diagrams on the far right illustrate the presence or absence of canonical motifs (CP, QFP, T, 1, 2, A, B′, C, D and E) on TERT AS protein variants drawn to scale. (PDF 774 kb) [file 12862_2017_949_MOESM11_ESM.pdf]

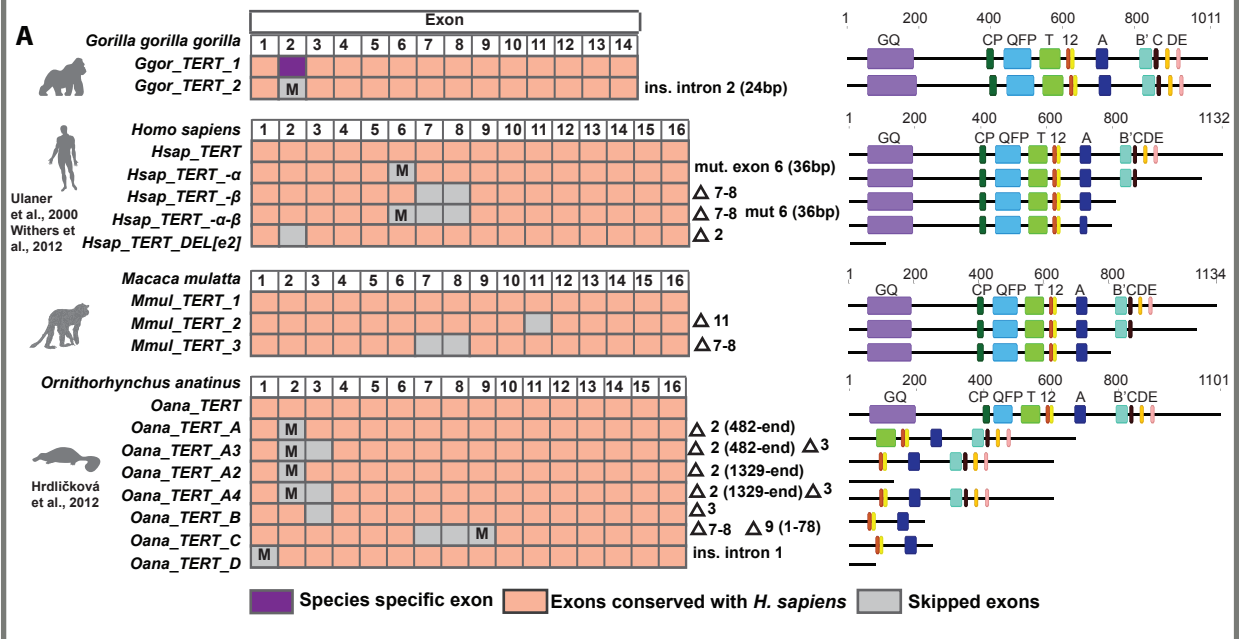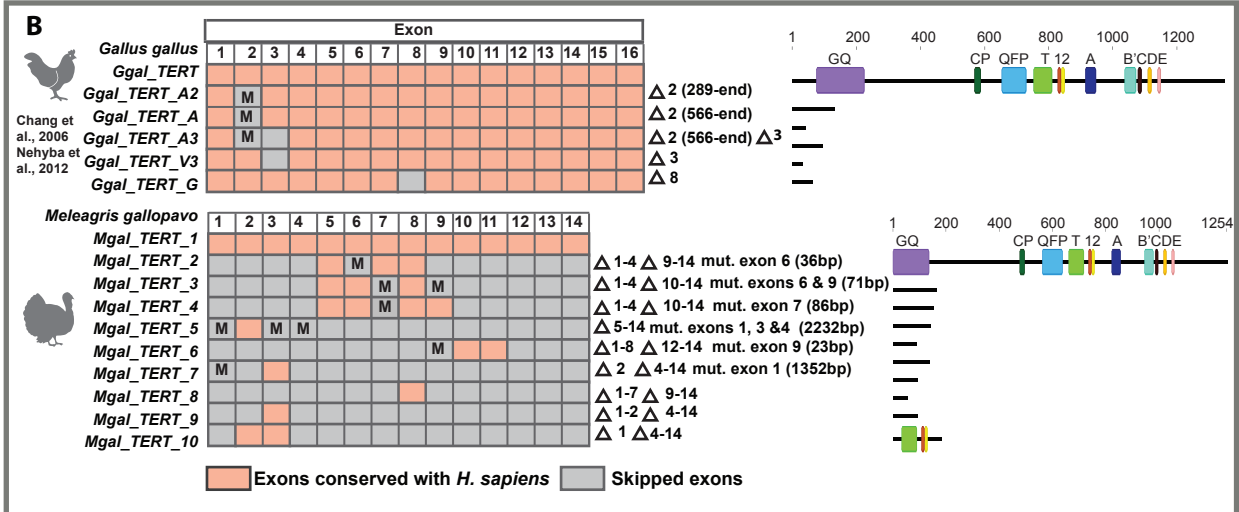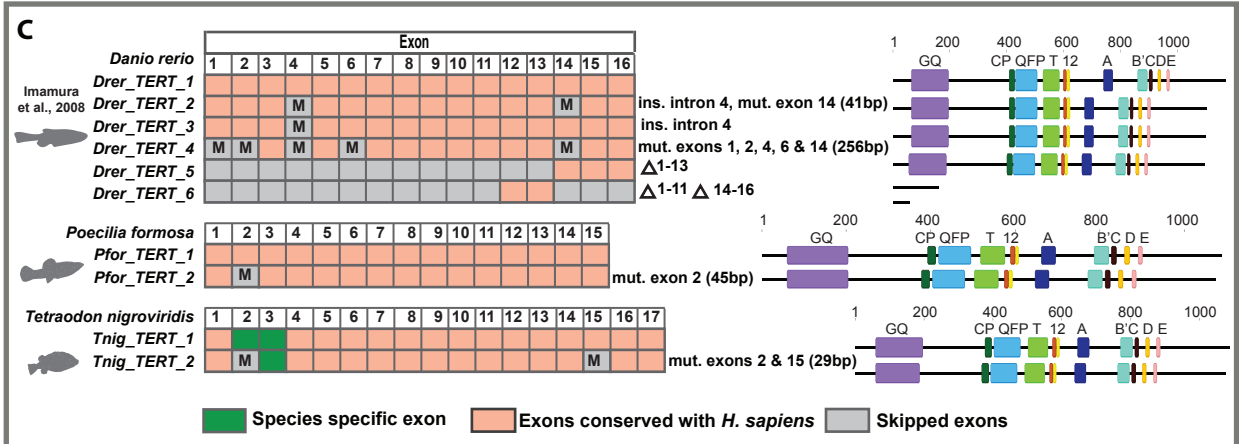

Supplement: Supplementary file 13 — TERT alternative splicing in vertebrates. AS variants are shown for (A) mammals, (B) birds and (C) fishes. Only the main AS variants for hTERT and chicken TERT are shown in this diagram. Additional variants have been reported in Hrdličková et al. [93]. The presence of AS variants with skipped exon 2 is conserved across different vertebrate species. AS variants with spliced exons 7 to 8 are conserved in mammals except in G. gorilla gorilla. Mammalian TERT has 16 exons whereas TERTs in non-mammalian vertebrates have anywhere between 14 to 17 exons. TERT AS variants data was obtained from Ensembl Genomes or NCBI. These are generated by computational predictions based on RNA sequencing read evidence. Schematic diagram depicts gene structure and splicing of TERT. Orange boxes represent exons conserved with hTERT. Purple and green boxes represent species-specific exons. Grey boxes represent skipped or deleted exons resulted from alternative splicing events. ‘M’ denotes splice site mutations, deletions or intron retention. The left margin shows TERT gene and AS variant names for each species and the right margin shows descriptive names of TERT AS sequences. The numbers in parentheses represent the length of splice site mutations (deletion or intron retention) for the respective AS variants except for platypus (O. anatinus) and chicken (G. gallus). For these two species, the numbers in parentheses represent the position of deletion in the exon indicated. Schematic diagrams on the far right illustrate the presence or absence of canonical motifs (GQ, CP, QFP, T, 1, 2, A, B′, C, D and E) on TERT AS protein variants drawn to scale. (PDF 334 kb) [file 12862_2017_949_MOESM13_ESM.pdf]

**A**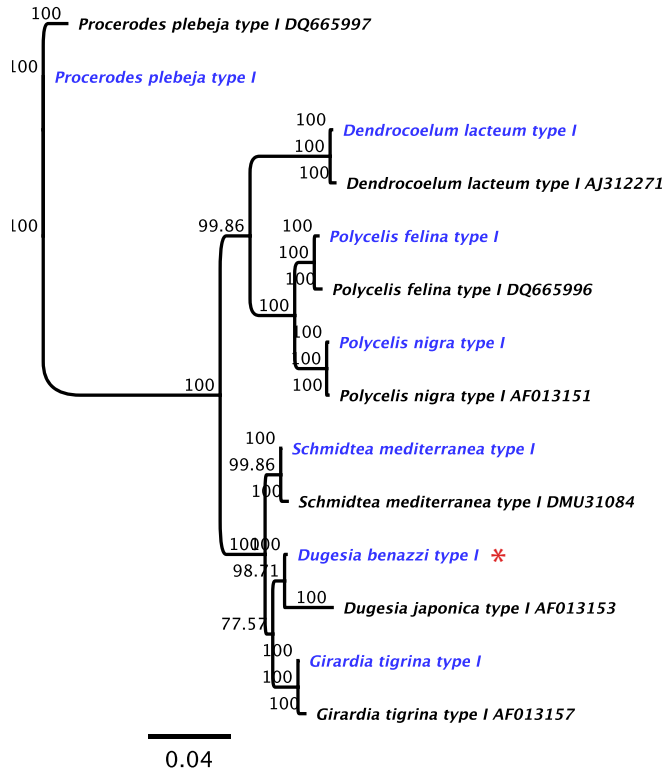**B**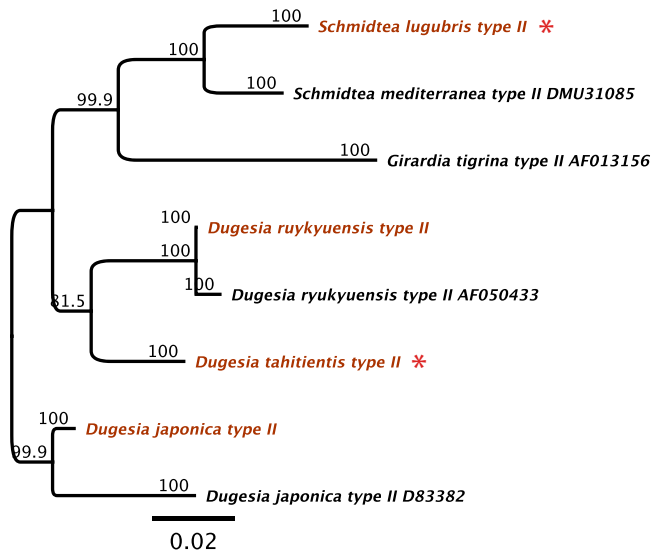

Supplement: Supplementary file 15 — Phylogenetic tree illustrating the positions of planarian type I and type II sequences in the 18S rDNA phylogeny. Sequences of 18S rDNA were cloned from 11 flatworm species in the lab for comparison against published sequences in GenBank. (A) Type I 18S rDNA sequences with species marked in blue as cloned in the lab. (B) Type II 18S rDNA sequences with species marked in brown as cloned in the lab. Asterisks indicate novel 18S rDNA sequences obtained from this study. (PDF 139 kb) [file 12862_2017_949_MOESM15_ESM.pdf]

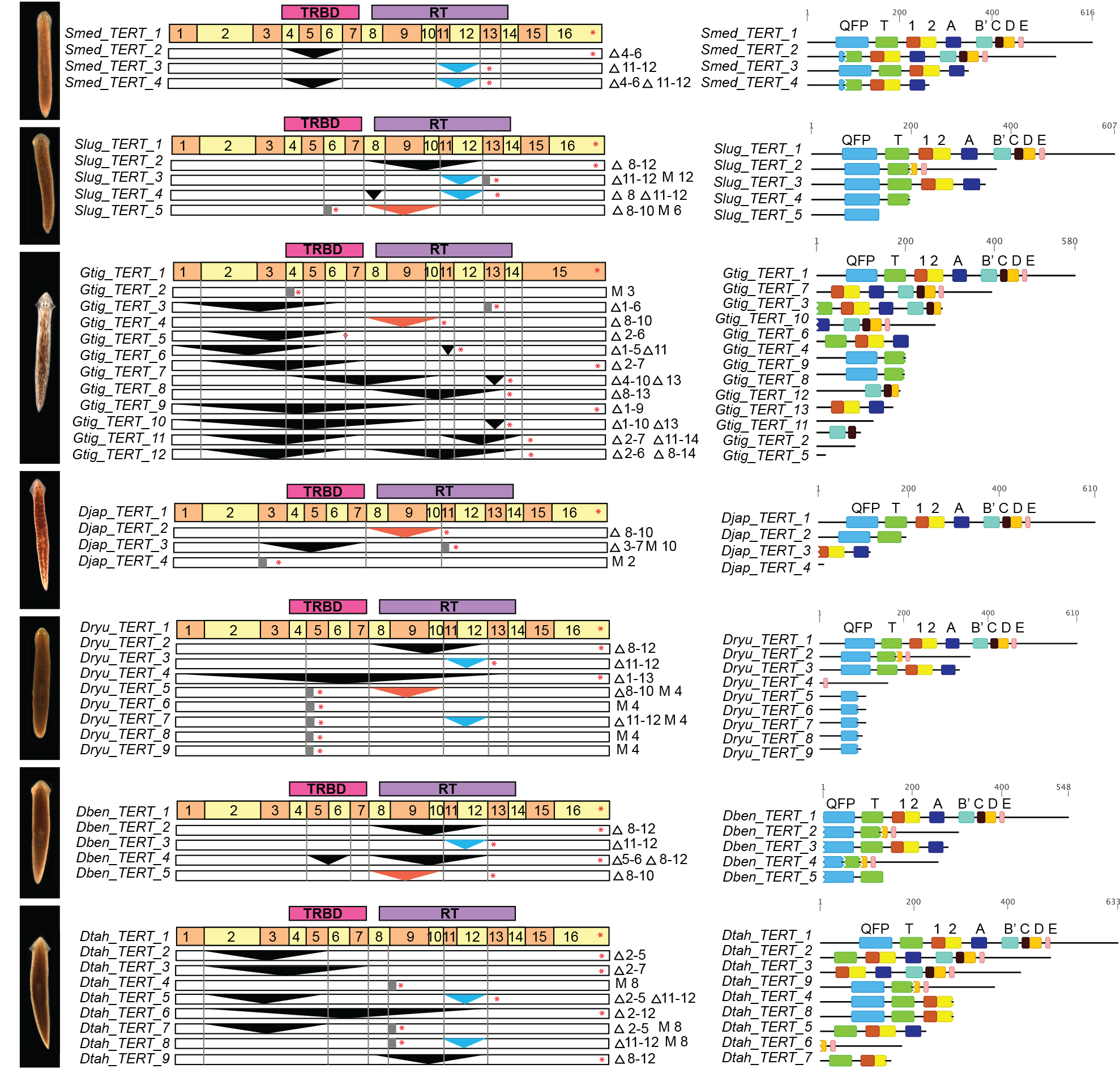

Supplement: Supplementary file 17 — TERT in Dugesiidae is alternatively spliced. Comparison of alternatively spliced (AS) variants in seven Dugesiidae species from the genera Schmidtea, Girardia and Dugesia. Structure of AS variants in S. mediterranea, S. lugubris, G. tigrina, D. japonica, D. ryukyuensis, D. benazzi and D. tahitientis. Full-length or wild-type TERT structure is shown at the top of each set of AS variants. Functional TERT domains (TRBD and RT) and positions of (putative) exons are indicated. Deletions (skipped exons) are denoted by triangles and insertions (retained introns or splice site mutations) are denoted by grey rectangles. ‘M’ abbreviations represent splice site mutations. Red triangles represent conserved alternatively spliced exons in all Dugesiidae species except S. mediterranea and D. tahitientis. Blue triangles represent conserved alternatively spliced exons in all Dugesiidae species except G. tigrina and D. japonica. Asterisks indicate stop codon positions caused by frame shift mutation or retained introns. The left margin shows TERT gene and AS variant names for each species and the right margin shows descriptive names of TERT AS sequences. Schematic diagrams on the far right illustrate the presence or absence of canonical motifs (QFP, T, 1, 2, A, B′, C, D and E) on TERT AS protein variants drawn to scale. (JPEG 1654 kb) [file 12862_2017_949_MOESM17_ESM.jpg]

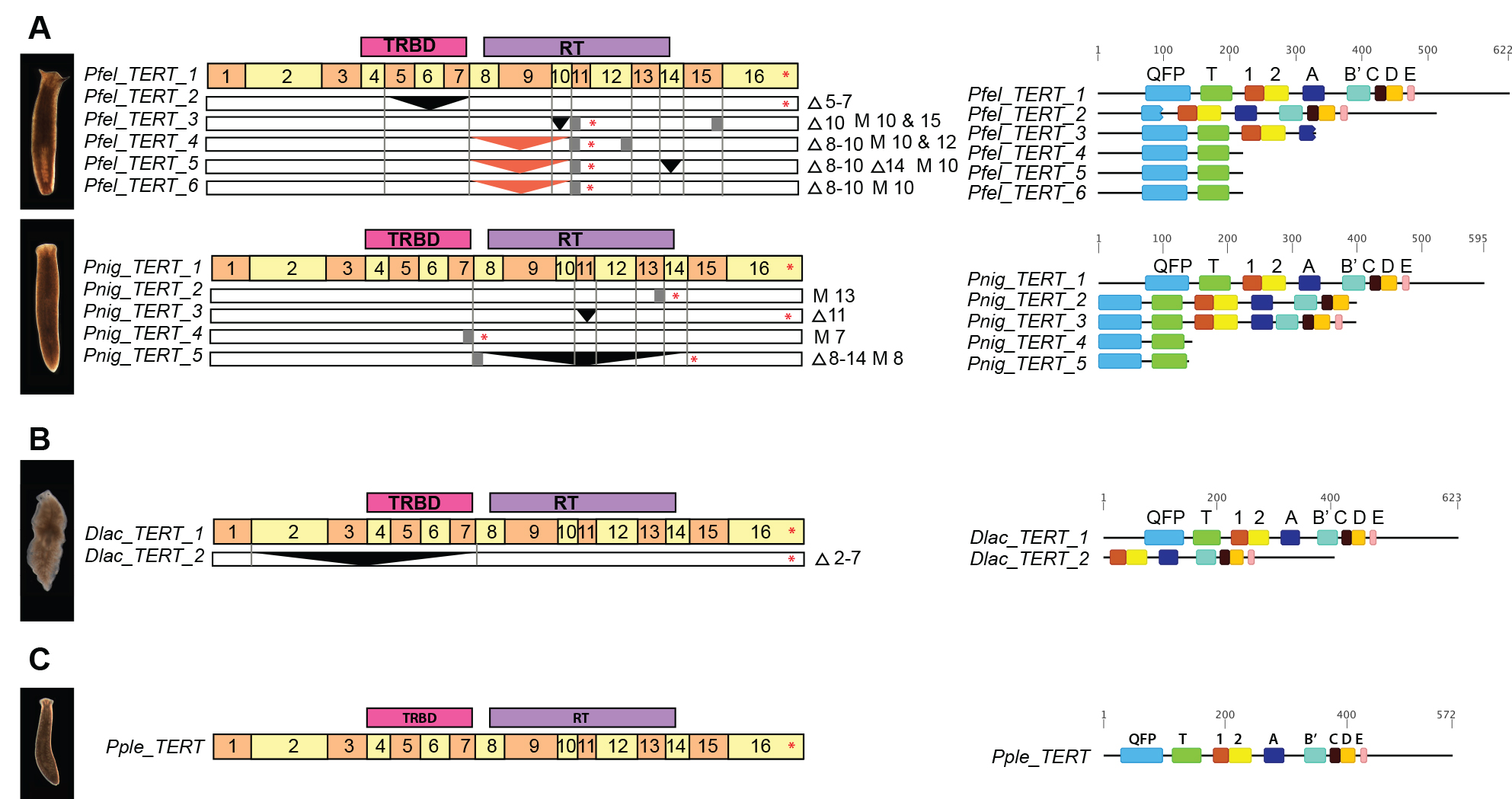

Supplement: Supplementary file 18 — TERT in Planariidae and Dendrocoelidae but not Procerodidae is alternatively spliced. Comparison of AS variants in two Planariidae species from the genus Polycelis, one Dendrocoelidae species and one Procerodidae species. Structure of AS variants in (A) Planariidae P. felina and P. nigra, (B) Dendrocoelidae D. lacteum and (C) Procerodidae P. plebeja. Genomic sequence data is not available for these flatworm species. Therefore, putative TERT exon-intron boundaries were annotated based on Smed_TERT sequence and confirmed by AS variant analyses of each species. Full-length or wild-type TERT structure is shown at the top of each set of AS variants. Functional TERT domains (TRBD and RT) and positions of (putative) exons are indicated. Deletions (skipped exons) are denoted by triangles and insertions (retained introns or splice site mutations) are denoted by gray rectangles. Red triangles represent alternatively spliced exons conserved with Dugesiidae. Asterisks indicate stop codon positions caused by frame shift mutation or retained introns. The left margin shows TERT gene and AS variant names for each species and the right margin shows descriptive names of TERT AS sequences. Schematic diagrams on the far right illustrate the presence or absence of canonical motifs (QFP, T, 1, 2, A, B′, C, D and E) on TERT AS protein variants drawn to scale. (JPEG 673 kb) [file 12862_2017_949_MOESM18_ESM.jpg]

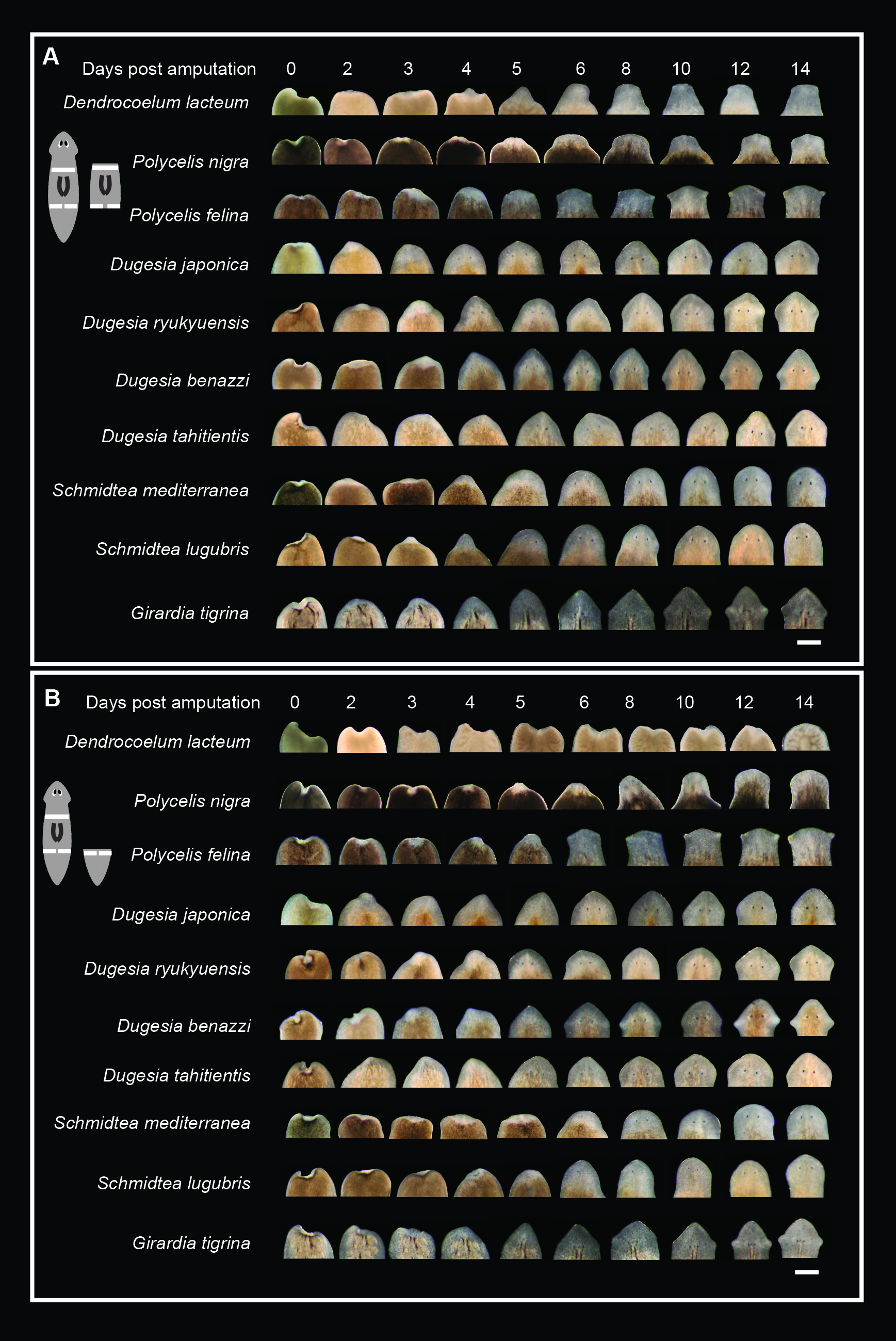

Supplement: Supplementary file 21 — Regenerative capacity in planarians. Transverse sections assaying regenerative abilities along anterior-posterior axis of planarians. Ten worms were used for each species and were amputated into three fragments each (head, trunk and tail). Representative images depict time-course observation of the same animal undergoing cephalic regeneration in (A) trunk and (B) tail fragments. Anterior blastema (unpigmented region) was imaged. Appearances of pigmented eyes were noted and plotted as frequency graphs. Scale bars indicate 0.5 mm. (JPEG 3354 kb) [file 12862_2017_949_MOESM21_ESM.jpg]

**A**

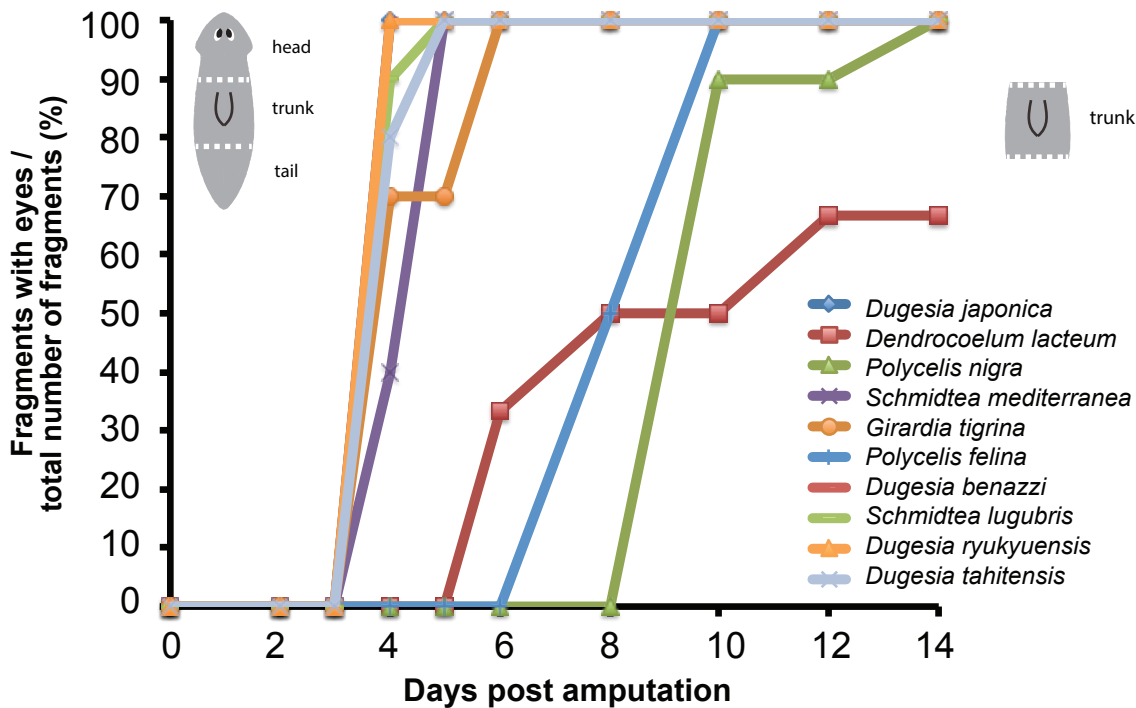

# B

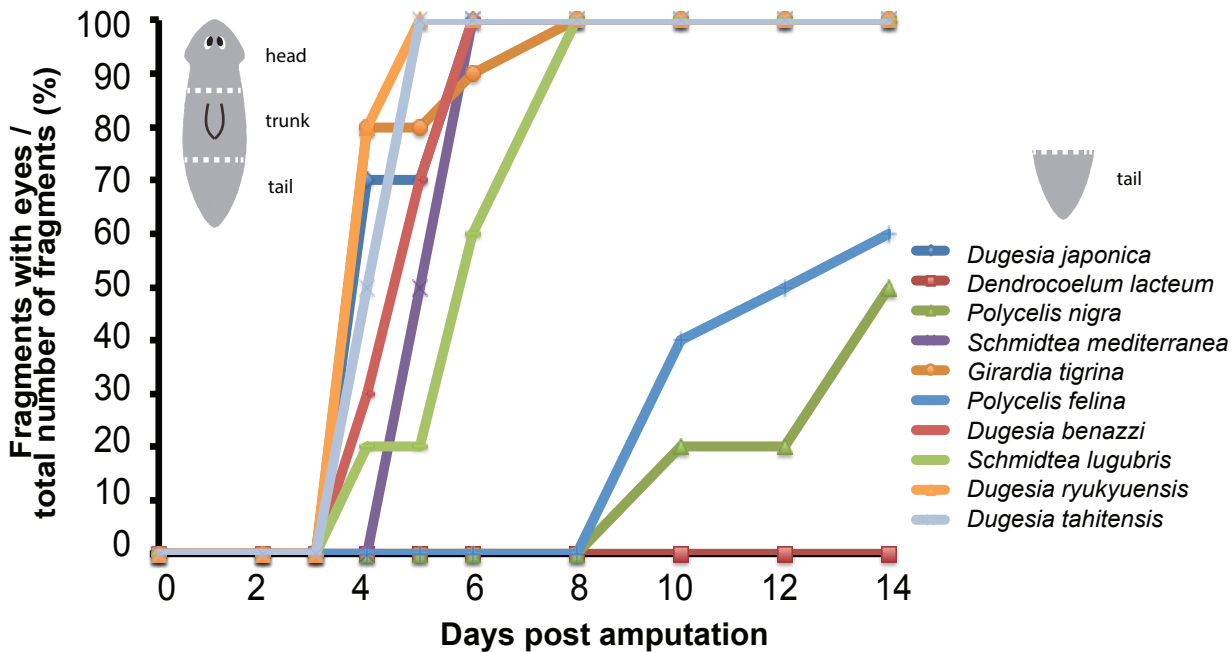

Supplement: Supplementary file 22 — Head regeneration frequency graph for (A) trunk fragments and (B) tail fragments of amputated flatworms (N = 10 for each species). Cartoon depicts the amputation planes used. The timing of eye appearance in cohorts of 10 fragments was noted. P. plebeja failed to regenerate at all and is not shown. (PDF 182 kb) [file 12862_2017_949_MOESM22_ESM.pdf]
