# Supplementary material for: The protein subunit of telomerase displays patterns of dynamic evolution and conservation across different metazoan taxa
Source: BMC Evol Biol. 2017 Apr 26;17:107. doi: 10.1186/s12862-017-0949-4 (PMC5405514; doi:10.1186/s12862-017-0949-4)
Supplement: Supplementary file 12 — TERT alternative splicing in mollusc. Schematic diagram depicts gene structure and TERT AS variants. Yellow boxes represent Aplysia californica exons. Gray boxes represent skipped exons resulted from alternative splicing events. ‘M’ denotes splice site mutations, deletions or intron retention. The left margin shows TERT gene and AS variant names for each species and the right margin shows descriptive names of TERT AS sequences. The numbers in parentheses represent the length of splice site mutations (deletion or intron retention) for the respective AS variants. Schematic diagrams on the far right illustrate the presence or absence of canonical motifs (QFP, T, 1, 2, A, B′, C, D and E) on TERT AS protein variants drawn to scale. (PDF 178 kb) [file 12862_2017_949_MOESM12_ESM.pdf]

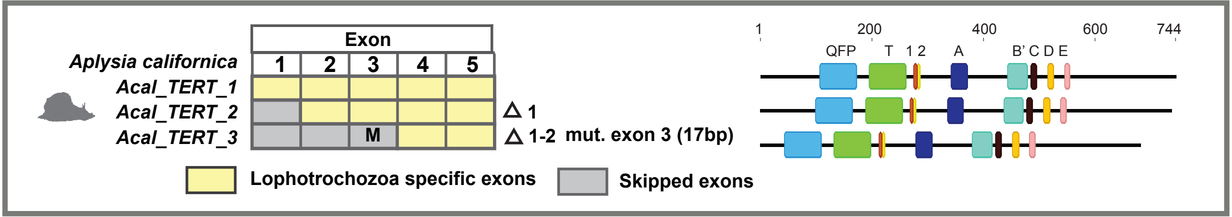
Exon
Aplysia californica
1
2
3
4
5
Acal_TERT_1
Acal_TERT_2
Acal_TERT_3
M
△ 1
△ 1-2 mut. exon 3 (17bp)
Lophotrochozoa specific exons
Skipped exons
1
200
400
600
744
QFP
T
1
2
A
B'
C
D
E
