# Supplementary material for: The protein subunit of telomerase displays patterns of dynamic evolution and conservation across different metazoan taxa
Source: BMC Evol Biol. 2017 Apr 26;17:107. doi: 10.1186/s12862-017-0949-4 (PMC5405514; doi:10.1186/s12862-017-0949-4)
Supplement: Supplementary file 53 — Schematic diagram demonstrating the annotation of intron positions in pairwise alignments with the hTERT sequence as a reference. Amino acid residues are highlighted in black (amino acids that are conserved amongst all sequences), grey (amino acids with similar properties) and light grey (amino acids that are dissimilar). (PDF 134 kb) [file 12862_2017_949_MOESM53_ESM.pdf]

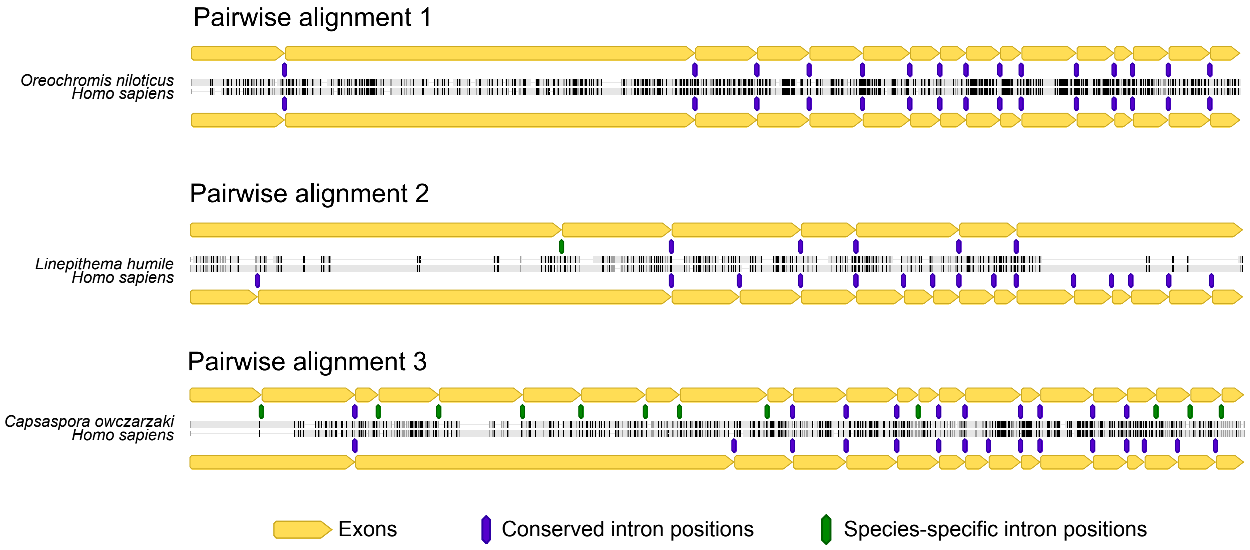
Pairwise alignment 1
Oreochromis niloticus
Homo sapiens
Pairwise alignment 2
Linepithema humile
Homo sapiens
Pairwise alignment 3
Capsaspora owczarzaki
Homo sapiens
Exons
Conserved intron positions
Species-specific intron positions
