# Supplementary material for: The protein subunit of telomerase displays patterns of dynamic evolution and conservation across different metazoan taxa
Source: BMC Evol Biol. 2017 Apr 26;17:107. doi: 10.1186/s12862-017-0949-4 (PMC5405514; doi:10.1186/s12862-017-0949-4)
Supplement: Supplementary file 3 — Multiple sequence alignments of the (A) TEN domain (GQ motif) and TRBD domain (CP, QFP, T) and (B) RT domain (1, 2, A, B′, C, D and E) from unicellular relatives of metazoans. Areas highlighted in coloured boxes delimit the canonical motifs. Boxes also denote species- or class-specific motifs with their respective descriptions written inside. Except for Capsaspora, the protozoan orders are represented by colour codes in the figure inset. (PDF 2723 kb) [file 12862_2017_949_MOESM3_ESM.pdf]

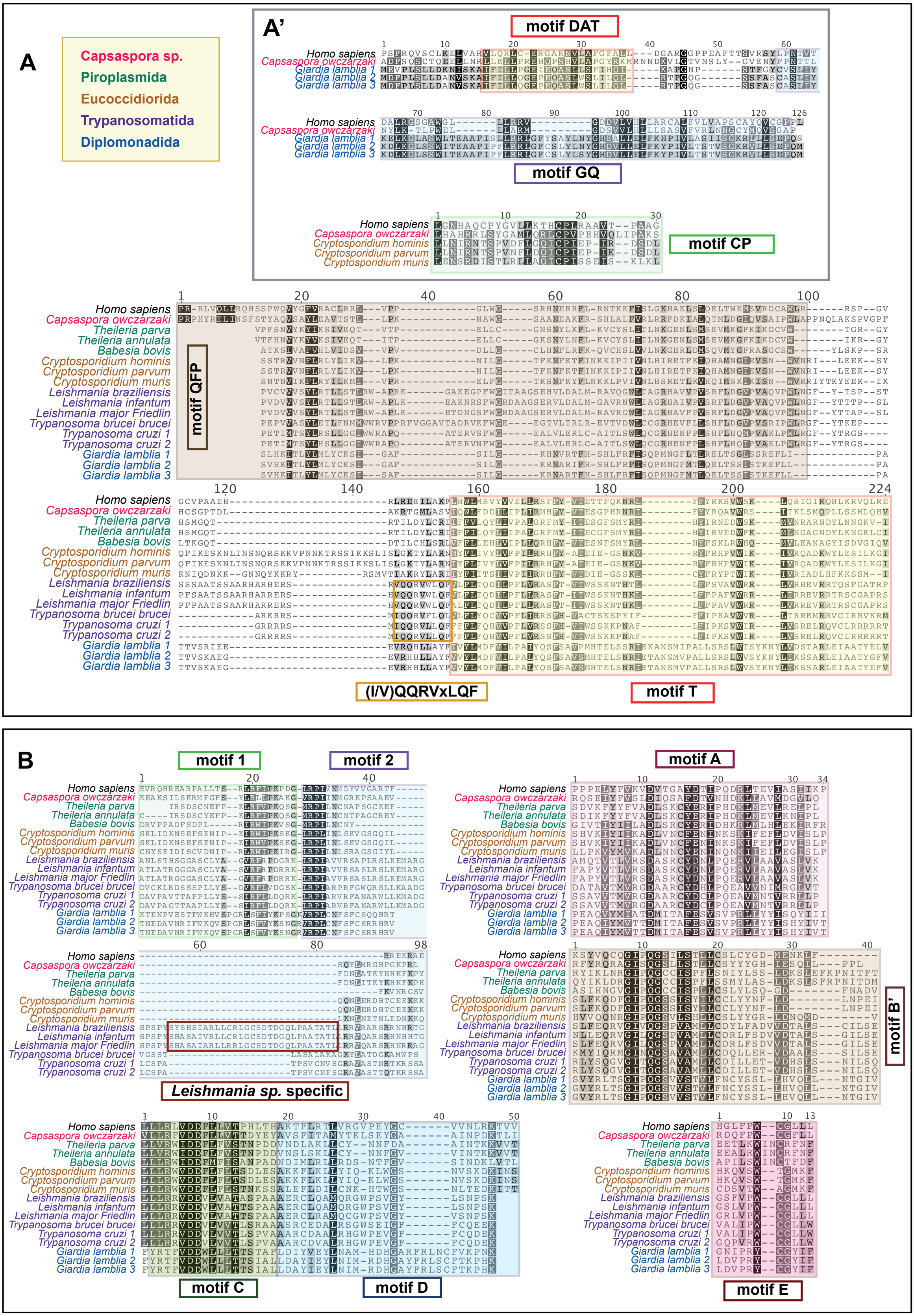

A
A’
Capsaspora sp.
Piroplasmida
Eucoccidiorida
Trypanosomatida
Diplomonadida
motif DAT
motif GQ
motif CP
motif QFP
(I/V)QQRVxLQF
motif T
Homo sapiens
Capsaspora owczarzaki
Giardia lambia 1
Giardia lambia 2
Giardia lambia 3
Cryptosporidium hominis
Cryptosporidium parvum
Cryptosporidium muris
Theileria parva
Theileria annulata
Babesia bovis
Leishmania braziliensis
Leishmania infantum
Leishmania major Friedlin
Trypanosoma brucei brucei
Trypanosoma cruzi 1
Trypanosoma cruzi 2
B
motif 1
motif 2
motif A
motif B’
Leishmania sp. specific
motif C
motif D
motif E
