# Supplementary material for: The protein subunit of telomerase displays patterns of dynamic evolution and conservation across different metazoan taxa
Source: BMC Evol Biol. 2017 Apr 26;17:107. doi: 10.1186/s12862-017-0949-4 (PMC5405514; doi:10.1186/s12862-017-0949-4)
Supplement: Supplementary file 4 — Multiple sequence alignments of the (A) GQ and CP motifs and (B) T motif and RT domain (1, 2, A, B′, C, D and E) from nematodes. Areas highlighted in coloured boxes delimit the canonical motifs. Boxes also denote Species- or Class-specific motifs with their respective descriptions written inside. Nematode classes are represented by colour codes in the figure inset. (PDF 2291 kb) [file 12862_2017_949_MOESM4_ESM.pdf]

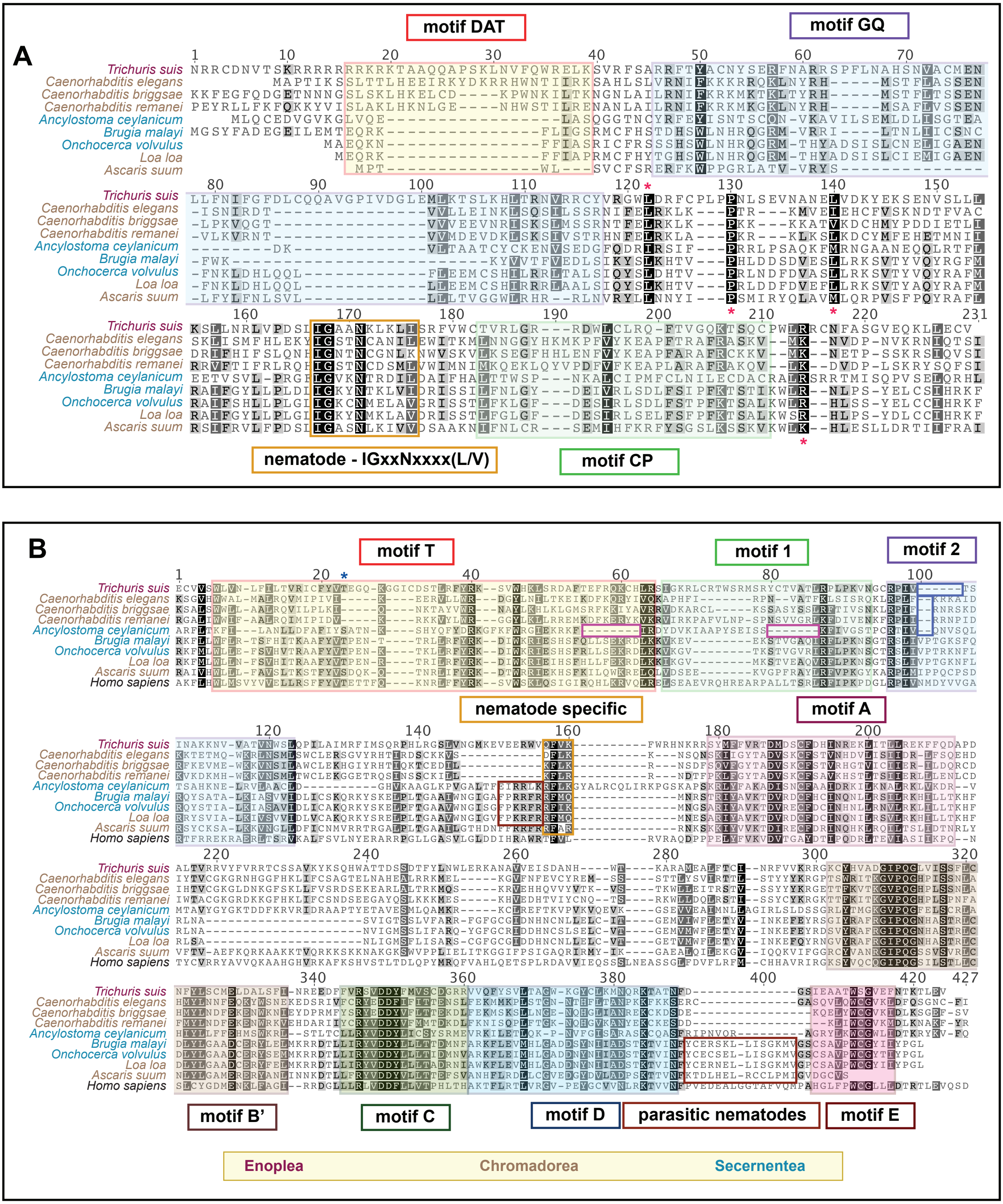
A
motif DAT
motif GQ
Trichuris suis
Caenorhabditis elegans
Caenorhabditis briggsae
Caenorhabditis remanei
Ancylostoma ceylanicum
Brugia malayi
Onchocerca volvulus
Loa loa
Ascaris suum
nematode - IGxxNxxxx(L/V)
motif CP
B
motif T
motif 1
motif 2
Homo sapiens
nematode specific
motif A
motif B'
motif C
motif D
parasitic nematodes
motif E
Enoplea
Chromadorea
Secernentea
