# Supplementary material for: The protein subunit of telomerase displays patterns of dynamic evolution and conservation across different metazoan taxa
Source: BMC Evol Biol. 2017 Apr 26;17:107. doi: 10.1186/s12862-017-0949-4 (PMC5405514; doi:10.1186/s12862-017-0949-4)
Supplement: Supplementary file 8 — Multiple sequence alignments of the N-terminal linker regions from (A) early-branching metazoans, (B) crustaceans, (C) unicellular relatives of metazoans, (D) insects, (E) molluscs and (F) vertebrates. Boxes denote phylum-, order- or class-specific motifs with their respective descriptions written inside. (PDF 2999 kb) [file 12862_2017_949_MOESM8_ESM.pdf]

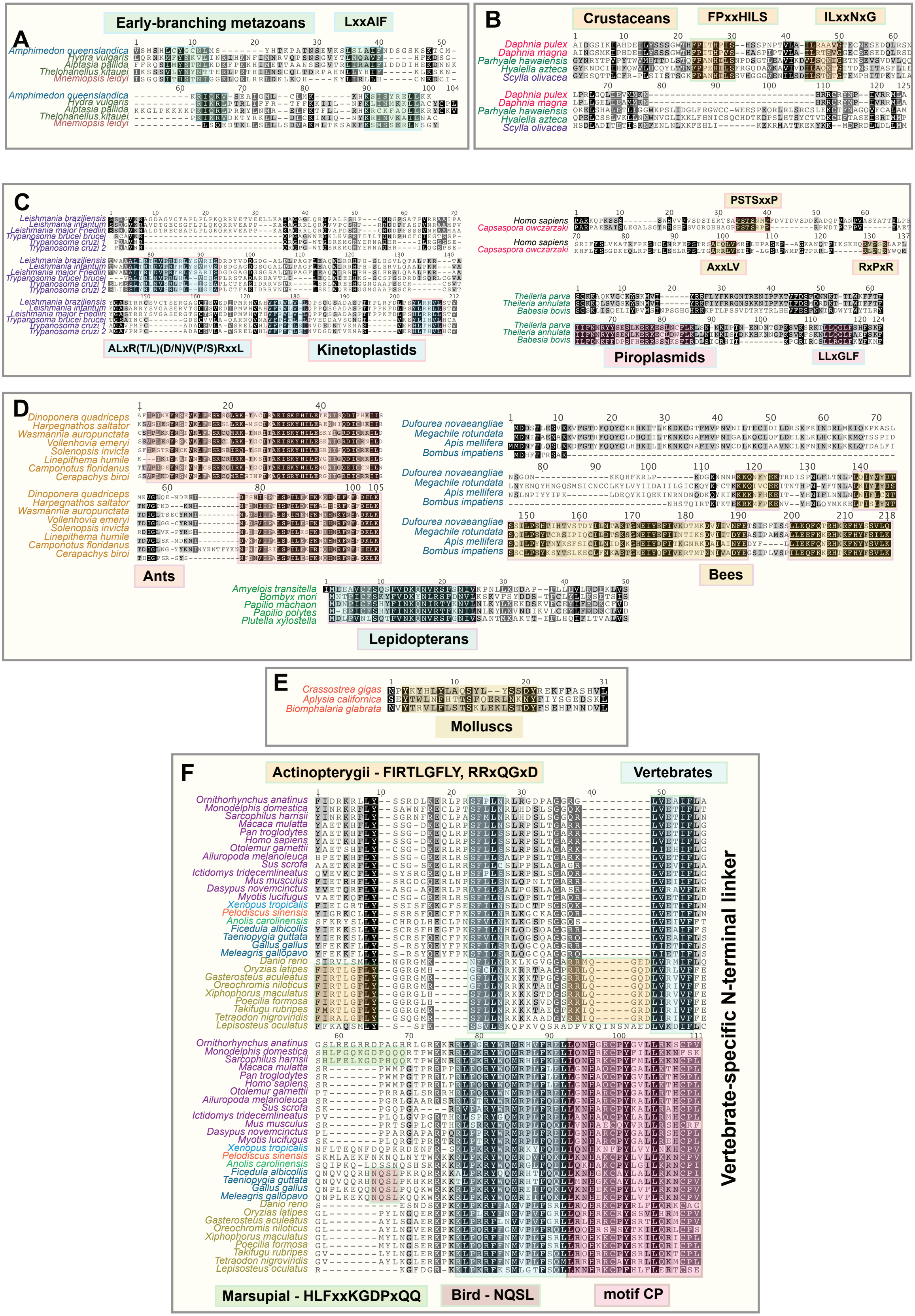
A
Early-branching metazoans
LxxAIF
Amphimedon queenslandica
Hydra vulgaris
Aiptasia pallida
Thelohanellus kitauei
Mnemiopsis leidyi
B
Crustaceans
FPxxHILS
ILxxNxG
Daphnia pulex
Daphnia magna
Parhyale hawaiensis
Hyalella azteca
Scylla olivacea
C
Leishmania braziliensis
Leishmania infantum
Leishmania major Friedlin
Trypanosoma brucei brucei
Trypanosoma cruzi 1
Trypanosoma cruzi 2
ALxR(T/L)(D/N)V(P/S)RxxL
Kinetoplastids
PSTSxxP
Homo sapiens
Capsaspora owczarzaki
AxxLV
RxPxR
Theileria parva
Theileria annulata
Babesia bovis
Piroplasmids
LLxGLF
D
Dinoponera quadriceps
Harpegnathos saltator
Wasmannia auropunctata
Vollenhovia emeryi
Solenopsis invicta
Linepithema humile
Camponotus floridanus
Cerapachys biroi
Ants
Dufourea novaeangliae
Megachile rotundata
Apis mellifera
Bombus impatiens
Bees
Amyelois transitella
Bombyx mori
Papilio machaon
Papilio polytes
Plutella xylostella
Lepidopterans
E
Crassostrea gigas
Aplysia californica
Biomphalaria glabrata
Molluscs
F
Actinopterygii - FIRTLGFLY, RRxQGxD
Vertebrates
Ornithorhynchus anatinus
Monodelphis domestica
Sarcophilus harrisii
Macaca mulatta
Pan troglodytes
Homo sapiens
Otolemur garnettii
Ailuropoda melanoleuca
Sus scrofa
Ictidomys tridecemlineatus
Mus musculus
Dasypus novemcinctus
Myotis lucifugus
Xenopus tropicalis
Pelodiscus sinensis
Anolis carolinensis
Ficedula albicollis
Taeniopygia guttata
Gallus gallus
Meleagris gallopavo
Danio rerio
Oryzias latipes
Gasterosteus aculeatus
Oreochromis niloticus
Xiphophorus maculatus
Poecilia formosa
Takifugu rubripes
Tetraodon nigroviridis
Lepisosteus oculatus
Vertebrate-specific N-terminal linker
Marsupial - HLFxxKGDPxQQ
Bird - NQSL
motif CP
