# Supplementary material for: The protein subunit of telomerase displays patterns of dynamic evolution and conservation across different metazoan taxa
Source: BMC Evol Biol. 2017 Apr 26;17:107. doi: 10.1186/s12862-017-0949-4 (PMC5405514; doi:10.1186/s12862-017-0949-4)
Supplement: Supplementary file 16 — Alignment of canonical motifs of TERT from 11 planarian species, three molluscs and hTERT. These include TRBD domain (QFP and T motifs) and canonical reverse transcriptase motifs (1, 2, A, B′, C, D and E). Clearly defined GQ and CP motifs were not identified from planarian TERTs. (PDF 1901 kb) [file 12862_2017_949_MOESM16_ESM.pdf]

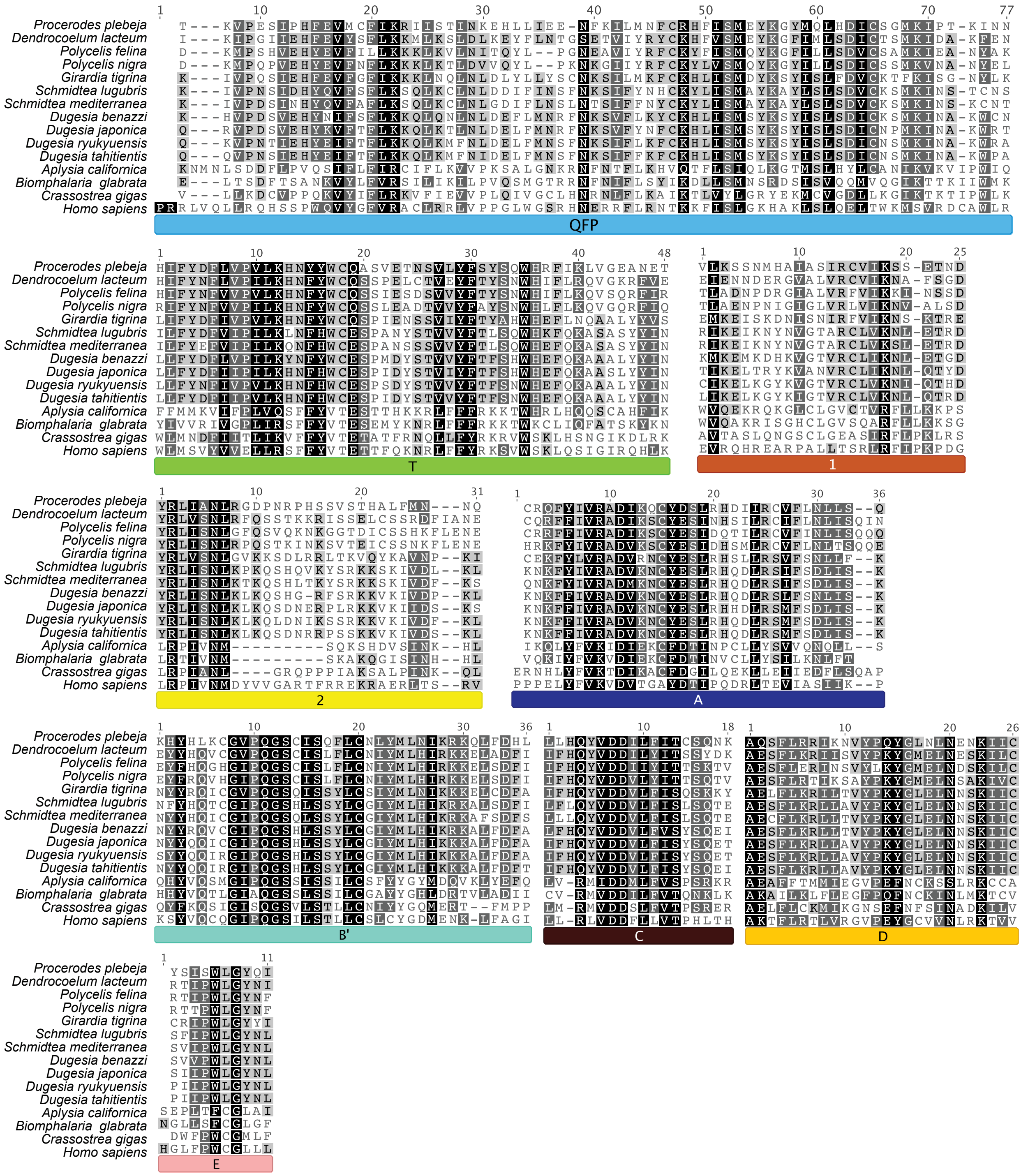
Procerodes plebeja
Dendrocoelum lacteum
Polycelis felina
Polycelis nigra
Girardia tigrina
Schmidtea lugubris
Schmidtea mediterranea
Dugesia benazzi
Dugesia japonica
Dugesia ryukyuensis
Dugesia tahitientis
Aplysia californica
Biomphalaria glabrata
Crassostrea gigas
Homo sapiens
QFP
T
1
2
A
B'
C
D
E
